# Differential effects of Mediterranean vs. Western diets on coronary atherosclerosis and peripheral artery transcriptomics

**DOI:** 10.3389/fnut.2025.1564741

**Published:** 2025-07-10

**Authors:** Aya Jamal Abusheikha, Corbin S. C Johnson, Noah Snyder-Mackler, Kip D. Zimmerman, Jacob D. Negrey, Kenneth L. Chiou, Brett M. Frye, Timothy D. Howard, Carol A. Shively, Thomas C. Register

**Affiliations:** ^1^Department of Pathology/Comparative Medicine, Wake Forest University School of Medicine, Winston-Salem, NC, United States; ^2^Department of Neurology, University of Washington, Seattle, WA, United States; ^3^Center for Evolution and Medicine, Arizona State University, Tempe, AZ, United States; ^4^School of Life Sciences, Arizona State University, Tempe, AZ, United States; ^5^School of Human Evolution and Social Change, Arizona State University, Tempe, AZ, United States; ^6^Center for Precision Medicine, Wake Forest University Health Sciences, Winston-Salem, NC, United States; ^7^Department of Molecular Medicine/Internal Medicine, Wake Forest University School of Medicine, Winston-Salem, NC, United States; ^8^School of Anthropology, University of Arizona, Tucson, AZ, United States; ^9^Department of Biology, Emory and Henry College, Emory, VA, United States; ^10^Department of Biochemistry, Wake Forest University School of Medicine, Winston-Salem, NC, United States

**Keywords:** iliac artery, carotid artery, atherosclerosis, Mediterranean diet, Western diet, nonhuman primates, social status, arterial transcriptome

## Abstract

Western diets and social subordination are associated with increased risk of cardiovascular disease. In this study, we investigated the impact of Western versus Mediterranean diets and social status on atherogenesis and arterial transcriptional profiles in a 30-month randomized study in middle-aged, cynomolgus monkeys (*Macaca fascicularis*). Atherosclerosis (intimal area) in the left anterior descending (LAD) coronary artery was higher in the Western diet group compared to the Mediterranean diet group (*F* = 5.25, *p* = 0.03). There was no effect of diet on intimal lesion size in the iliac and carotid arteries (*p* > 0.05). Diet altered the transcriptome in iliac arteries; at an FDR threshold of 0.05, seven transcripts were upregulated (*WDR62, PKDCC, SLC29A2, MARS1, RAD21L1, MAMDC4*, and ENSMFAG00000052859), and 13 transcripts were downregulated (*PIK3R1, PABPC1, PAQR8, ZNF667, FGGY, EIF4B, ALDH3A2, ANP32A, KDM3B, XPO7, RPS20*, *TOMM20*, and *CHCHD7*) in the Western compared to the Mediterranean diet cohort. These genes are associated with endothelial dysfunction, smooth muscle proliferation and migration, angiogenesis, and abnormal extracellular matrix (ECM) dynamics. In addition, two transcripts (ENSMFAG00000064154 [LncRNA] and ENSMFAG00000057515 [small nucleolar RNA U13]) were downregulated in subordinate monkeys relative to their dominant counterparts (FDR < 0.05). There was no effect of diet on the carotid artery transcriptome, but we did identify significant social status effects: Eleven transcripts were upregulated (*KCNQ4, STIM1, TNKS1BP1, CSNK1D, INPPL1, PNPLA7, F10, RAD9A*, KCNIP3, ENSMFAG00000059809 [LncRNA], and ENSMFAG00000053865 [secreted protein A0A7N9CS45]), and seven transcripts were downregulated (*IRAK1BP1, KIAA0513, SMIM15, PSMD14, TOPORS, ARPC2*, and ENSMFAG00000050714 [LncRNA]) in subordinate relative to dominant monkeys. These alterations were associated with dysregulated vascular tone and smooth muscle contractility, apoptosis, and abnormal ECM dynamics. These findings demonstrate differential effects of diet composition and social status depending on arterial sites. The effects of Western diet were observed primarily in the coronary and iliac arteries, whereas social status differences were observed primarily in the carotid arteries. Our results demonstrate that Western diets and social subordination have adverse, yet distinct and tissue-specific impacts on arterial atherogenesis and transcriptional profiles, highlighting the interplay between diet, social hierarchy, and vascular health.

## 1 Introduction

Dietary patterns play a pivotal role in human health, influencing various physiological processes and disease outcomes. Among dietary regimens, the Western and Mediterranean diets represent two contrasting paradigms with distinct compositions and health implications.

Western diets derive much of their protein and fat content from animal sources and are generally high in saturated fats, sodium, omega-6 (n-6) fatty acids, and processed simple sugars (1). Western diets have been shown to promote sympathetic nervous system arousal, insulin resistance, oxidative stress, and inflammation (2–4). Inflammation and oxidative stress contribute to the pathogenesis of atherosclerosis and poor cardiovascular health (5, 6). Western diets are also associated with increased risk of cardiometabolic diseases including obesity, hypertension, type 2 diabetes and coronary artery atherosclerosis (7–9). In contrast, Mediterranean diets derive most of their protein and fat from plant and fish sources, have higher proportions of monounsaturated, polyunsaturated omega-3 (n-3) fatty acids, and natural antioxidants; and have lower levels of refined simple sugars. Fruits and vegetables, which make a larger contribution to Mediterranean diets, are rich in antioxidants that help reduce oxidative stress and inflammation (6, 10, 11). Moreover, n-3 fatty acids (found in fish, nuts, and flaxseed) and polyphenols in olive oil possess anti-inflammatory and antioxidant properties (3, 11). The Mediterranean diet’s anti-inflammatory properties, attributed to its rich array of phytonutrients and n-3 fatty acids, may help protect against chronic diseases including cardiovascular disease (CVD) (12–15).

Diet may also adversely impact health through activation of stress mechanisms. In population studies, the Western diet pattern has been associated with greater perceived stress and higher sympathetic activity (8) and increased urinary cortisol levels (16); whereas the Mediterranean diet pattern has been associated with lower perceived stress (4, 17, 18) and cortisol levels (19). Psychological stress also promotes inflammation (20), and increases CVD risk (21–23). This raises the possibility that one of the pathways through which a healthy diet reduces CVD risk is by reducing psychological stress responses. This is especially pertinent in societies organized by social status hierarchies, as lower socioeconomic status (SES) has been associated with behavioral and physiological characteristics of stress (24, 25), as well as increased risk for atherosclerosis and CVD (26, 27).

Social determinants of health (SDHs), like SES and ethnicity, exhibit a significant impact on vascular diseases; in which SDHs contribute to disparities in disease prevalence, treatment accessibility, and health outcomes (28). Low SES correlates with higher disease prevalence and poorer postoperative outcomes, partly driven by limited healthcare access and lifestyle factors associated with lower-income settings (28). For example, individuals with lower SES face higher risks of peripheral artery disease and chronic venous disease, often attributed to occupational hazards prevalent in low-income employment sectors, that involves prolonged standing (29–31). Similarly, low SES populations in rural areas have limited access to specialized healthcare services, which further exacerbate health inequalities (32). Low SES is significantly associated with limited access to health care, as individuals with low incomes are more likely to be uninsured, experience poor-quality health care, and primarily seek medical attention during emergencies. Additionally, low-SES individuals often face limitations in care due to underfunded health facilities, contributing to poorer health outcomes (33).

SES significantly influences diet quality as well, in which individuals from low-SES groups typically consume poorer diet quality, characterized by lower amounts of fruits, vegetables, whole grains, fiber, and fish, and higher amounts of energy-dense, nutrient-poor processed foods (34, 35). These disparities are driven by economic limitations and reduced access to healthier food options, which are often more expensive and less available in low-income neighborhoods (36). Diet quality, assessed through the Dietary Quality Score (DQS), Nutrient Adequacy Ratio (NAR), and Dietary Diversity Score (DDS) were shown to consistently improve with increasing SES level (34). Another study showed that individuals of higher SES are significantly associated with greater food expenditure; thus, purchasing healthier food options (37).

On a physiological level, many studies explained the intricate relationships between biomarkers and SDHs in influencing the progression and outcomes of vascular diseases. Lower SES was shown to be significantly correlated with elevated levels of inflammatory biomarkers such as high-sensitivity C-reactive protein (hs-CRP) and interleukin-6 (IL-6) (38). Additionally, environmental factors like air pollution, prevalent in low-income areas, have been associated with increased matrix metalloproteinase levels, accelerating vascular damage and disease progression (39, 40). Moreover, social stressors linked to low SES, including limited education and healthcare access, exacerbate biological stress responses, further influencing disease severity through elevated cortisol and inflammatory markers (41). It is important to mention that it is difficult to separate the effects of low SES-related stress from the other factors associated with low SES in people, like air pollution, poor quality diet, and poor health care, that impact health. Therefore, a randomized preclinical trial eliminates most of these confounders.

Despite the substantial evidence supporting the health benefits of the Mediterranean diet, Western dietary patterns continue to prevail in many regions, contributing to the global burden of complex chronic diseases. Moreover, diet composition and its effect on physiological mechanisms have been mostly investigated in short term controlled trials which may not predict long term health effects (42). Long-term observational studies are confounded by other factors and behaviors (e.g., smoking behavior, alcohol consumption, physical activity levels), and rely on self-reported food intake (42–44). Long-term randomized feeding trials are thus required to better predict long term health effects. These relationships and uncertainties led us to design an experiment to address diet and behavior interactions in a rigidly controlled study in a well-established nonhuman primates (NHP) model of diet and psychosocial stress effects on health.

NHPs are useful models to study human health and complex diseases as they share many genetic, physiological, and behavioral phenotypes with humans, including key characteristics of energy metabolism, susceptibility to develop aging related diseases including metabolic and vascular disorders when consuming Western diets (45–49). Additionally, similar to humans, individual NHPs differ in their degree of social integration and isolation, in which multiple factors can affect sociality, including sex, age, social status, and kin networks (50, 51). NHPs can also participate reliably in long term trials with extensive phenotyping in controlled studies. In both wild and captive groups, female macaques exhibit social status hierarchies (1). Like humans, social hierarchies in macaques are associated with social inequalities in health. Similarities between NHPs and humans suggest that NHPs may share mechanisms linking social status and health with humans. The ability to directly record social behaviors in NHPs avoids the confounders found in human studies, thus supporting their use as a model system.

Subordinate monkeys (defined here as those in the lower half of the hierarchy) spend more time alone, receive more aggression, are groomed less, and are more fearful and anxious than their dominant counterparts (1, 47, 50, 52–54). Subordinates respond to a standardized stressor with a higher initial heart rate and recover more slowly than dominants (47, 50, 55). Subordinates also have higher visceral obesity, more inflammation, lower bone density (56, 57), and develop more diet-induced coronary and carotid artery atherosclerosis than their dominant counterparts (58, 59). With respect to social status and health, it is important to note that most of the available data from epidemiological studies and macaques has been derived from subjects consuming a Western diet (60, 61).

Here we report the results of our 30-month randomized trial, in which diet was manipulated, environment and confounders were controlled, and multiple cardiovascular tissues were collected at the end of the study. The purpose of the present study was to determine the effects of diet (Western versus Mediterranean) and social status on atherosclerosis in the coronary, iliac, and carotid arteries and to identify putative molecular mechanisms underlying the effects. We aimed to determine the transcriptome-related effects of diet and social status in the iliac arteries, which are a good surrogate for coronary arteries in macaques (49, 62, 63), and the carotid arteries, which are important in blood pressure control and associated with stroke risk (64). Our overall hypotheses were that the Mediterranean diet would promote overall vascular health and potentially protect against adverse effects of subordinate status.

## 2 Materials and methods

### 2.1 Subjects

Thirty-eight middle-aged (mean = 9.0, range = 8.2–10.4 years, estimated by dentition) female cynomolgus monkeys (*Macaca fascicularis*) were obtained from Shin Nippon Biomedical Laboratories US SRC (Alice, TX) and housed at the Wake Forest University School of Medicine (Winston-Salem, NC) (42). Based on previously published comparative aging models, a 9-year-old *Macaca fascicularis* is approximately equivalent to a 35–40-year-old human female in terms of physiological and reproductive aging (e.g., age at menarche, menopause, and onset of age-related pathologies) (65). Monkeys were quarantined in single cages for 1 month, then socially housed in groups of 3–4 animals in indoor enclosures (3 m x 3 m x 3 m), maintained on a 12/12 light/dark cycle with exposure to daylight, and water *ad libitum*. All animal manipulations were performed according to the guidelines of state and federal laws, the US Department of Health and Human Services, and the Animal Care and Use Committee of Wake Forest University School of Medicine.

### 2.2 Design

The study design was a preclinical randomized trial 39 months in length. During an 8-month baseline phase, the animals consumed standard monkey chow (Supplementary Table 1; monkey diet 5037/5038; LabDiet, St. Louis, Missouri) with *ad libitum* access to water. Monkeys were allowed to adapt to their social groups for 3 months followed by assessments of behavior, physiology, and social status during the last 4 months of the baseline phase. Individual social groups were then assigned to receive a Western diet (Western *n* = 21) or a Mediterranean diet (Mediterranean *n* = 17) by stratified randomization to balance body mass index, basal plasma cortisol and triglyceride concentrations (42). Comprehensive physical, behavioral, and physiological characteristics were assessed during the diet intervention phase.

### 2.3 Experimental diets

Western and Mediterranean diets were formulated to be matched on proportions of calories from macronutrients (protein, carbs, and fats), cholesterol content (~ 320 mg / 2000 calories/day), and caloric density (2.8 cal/g wet diet; Supplementary Table 1). Diet composition was designed and prepared at the Wake Forest School of Medicine Comparative Medicine Diet Lab. Western diet was designed to be similar to the nutritional composition of the typical North American diet, with protein and fat derived mainly from animal sources, the diet was high in saturated fats and low in mono- and poly-unsaturated (66). The Mediterranean diet derived most of the protein and fat from plant sources, and was relatively high in mono- and poly-unsaturated fatty acids (67). Further, the n-3: n-6 fatty acid ratio was approximately 5-fold higher in the Mediterranean diet compared to the Western diet (68).

### 2.4 Food consumption

Methods for monitoring food consumption have been previously described (42). Briefly, custom individual feeding cages were fabricated and placed inside group pens. Monkeys were trained to run into the feeding cages on voice command, consume their food, and then be released back into their group pen. Each monkey was offered 120 kcal diet/kg BW/day. Throughout the experiment, the animals finished feeding in the first 30 min. During the baseline phase and the first year of the treatment phase, the monkeys were fed for 2 h once a day at ~0730 h. During the last half of the treatment phase, beginning in month 16, one-half of the ration was fed at ~0730 h for 1 h, and the other half was fed at ~1,330 h for 1 h. Food consumption was estimated by weight before and after the feeding periods during month 5 of the baseline phase and months 3–6, 7–10, 17–20, and 27–30 of the experimental phase; average consumption was calculated for each monkey for each time period.

### 2.5 Quantification of social status

Social status rankings were determined throughout the experiment by recording the outcomes of agonistic interactions within monkeys of the same social group (47, 50, 69, 70). The highest-ranking monkey of each group was the female that defeated all others, as demonstrated by her ability to consistently elicit submissive responses. The female that defeated all but the first-ranking monkey was designated as the second-ranking monkey, etc. Monkeys formed relatively stable linear hierarchies, and social status relationships within groups were typically transitive, meaning that if monkey 1 was dominant over monkey 2, and monkey 2 was dominant over monkey 3; then monkey 1 was dominant over monkey 3 as well. We determined social status by defining the number of monkeys submissive to a given monkey, corrected for group size so the resulting relative rank could be compared across social groups (71). Accordingly, relative rankings varied from 0 to 1. For some analyses, we dichotomized social status, in which females with a relative rank greater than 0.5 were considered dominant and labeled as 1, and all others were considered subordinate (50, 72). Social subordination appears stressful, as subordinates in this study received more aggression, were groomed less, spent more time fearfully scanning, had higher systolic blood pressure, and had higher sympathetic nervous system activity than did dominants (73). Thus, we used social status as a proxy for psychosocial stress.

### 2.6 Behavioral characterization

Agonistic and affiliative behavior were recorded in 10 min focal animal observations (74) beginning in the third month of the experimental phase as previously described (73). Behavioral data were collected weekly during two 10 min focal observations, balanced for time of day, for 6 weeks during the baseline phase (2 h/monkey total) and for 14 months during the experimental phase (mean = 17.6 h/monkey total). Behaviors recorded included affiliative behaviors: percent of time spent alone (out of monkey’s arm’s reach), percent of time spent in body contact with other monkeys, and percent of time spent in close proximity (within monkey’s arm’s reach) (67, 75–77). Anxiety behavior observed is the frequency of scratching, itching, yawning, and self-grooming (67, 75–77). Percent of time spent depressed is defined by recording the time a monkey spends in a depressive behavior (slumped or collapsed body posture accompanied by a lack of responsivity to environmental events with eyes open) (78–81).

### 2.7 Arterial tissue collection and processing

At the end of the experimental phase, monkeys were anesthetized with pentobarbital (30–50 mg/kg) to obtain a surgical level of anesthesia, and we collected coronary, iliac and carotid arteries. The heart and coronary arteries were perfusion-fixed with 4% paraformaldehyde at 100 mm Hg for 1 h, followed by storage in 70% ethanol at 4°C. Twelve blocks (each 3 mm in length) were cut perpendicular to the long axis of the left circumflex (LCX), left anterior descending (LAD), and right coronary artery (RCA). Iliac and carotid arteries were subdivided perpendicular to long axis into 5 sections, sections 1,3, and 5 were fixed for assessment of lesion size, sections 2 and 4 were rinsed in ice-cold lactated Ringer’s solution and placed in RNAlater® for removal of adventitia and adherent connective tissue. Intima-media sections for transcriptomic analyses were then slam frozen and stored in a − 80°C freezer until processing.

### 2.8 Atherosclerosis measures

Blocks of coronary, carotid, and iliac arteries were embedded in paraffin, and 5 μm sections were stained with Verhoeff-van Gieson’s stain. Images were captured for analysis using ImageProPlus software, and cross-sectional area of the arterial intima was measured as described previously (63).

### 2.9 RNA extraction and processing

RNA was isolated from iliac and carotid arteries samples using the QIAGEN Quick-Start RNeasy Mini Kit (QIAGEN, Hilden, Germany). RNA integrity number (RIN) scores were determined to assess quality with an average score of 8 across samples. One iliac artery RNA sample with a RIN score of only 4.4 was excluded from all analyses, leaving a final sample size of 35 iliac arteries (*n* = 20 Western diet, *n* = 15 Mediterranean diet). We processed RNA for bulk-RNA sequencing by mapping 37,781 reads to the *Macaca fascicularis* reference genome (Macaca_fascicularis_6.0) using *STAR-2.5.2a* (82), then converted to a sample-by-gene read count matrix using *featureCounts* (83).

### 2.10 Quality control

Read counts data were filtered using the *rpkm* function from the *edgeR* R package (84), excluding genes with median reads per kilobase per million reads mapped (rpkm) of less than one, which resulted in 13,020 and 13,206 genes for downstream analyses in iliac and carotid arteries, respectively. Read counts were then normalized using the weighted trimmed mean of M-value (TMM) method (85) followed by log2 transformation using the *voom* function of the R package *limma* (86), which also applies normalization scaling factors that take into account the differences in sequencing depth. To control for technical variables related to RNA quality and reads mapping, residual gene expression was determined from a model of normalized gene expression as a function of the technical variables that significantly contribute to variation in gene expression. Principal component analysis (PCA) (87) was performed using the *prcomp* function in R to determine effects of technical variables. For the iliac artery, we controlled for library size, percent of uniquely mapped reads, and RNA concentration (ng/ul); for the carotid artery, we controlled for percent of uniquely mapped reads, RNA concentration (ng/ul), and RNA integrity. The resulting residual gene expression values (called the residual matrix) were used in the subsequent analyses. Hierarchal clustering analysis was carried out using the *hclust* function from the WGCNA R package (88) to identify similar groups and potential sample outliers.

### 2.11 Modeling differential gene expression by diet composition and social status

Differential expression analysis was employed to identify differences in transcript levels in response to diet, social status and potential interactions. The R package *EMMREML* was used to model the residual expression of each gene as a function of diet and/or social status with a linear mixed effects model controlling for relatedness among monkeys (89). We estimated genetic relatedness using the *ngsRelate* program (90) with SNP genotype inferred from the RNA-seq reads using *bcftools mpileup* (91). Social status contributions to outcomes were assessed using both a continuous (relative rank score) or dichotomous (dominant rank vs. subordinate rank) approach. We used the *qvalue* R package (92) to calculate the false discovery rates (FDR) for each gene, and reported genes that passed an FDR threshold of 0.05 as significant. To examine global patterns of variations in gene expression, principal component analysis was applied to the correlation matrix of normalized residual gene expression using the *prcomp* function in R.

### 2.12 Enrichment analysis and identifying biological pathways

Enrichment of biological processes and regulatory networks exhibiting diet-specific and social status-specific activation patterns were assessed using Pathway Enrichment Analyses. Pathway analysis was performed with QIAGEN’s Ingenuity Pathway Analysis (IPA, QIAGEN Redwood City).^1^ Given the limited number of differentially expressed transcripts (DETs) identified at FDR threshold of 0.05, we expanded our criteria to include genes with an FDR threshold of 0.2 (*n* = 174 for iliac arteries, *n* = 216 for carotid arteries).

### 2.13 Correlations between gene expression and phenotypic variables

During the last half of the experimental phase (13–31 months on experimental diet), study subjects underwent an extensive characterization of different phenotypes that represent categories of anatomy, physiology, and behavior. Detailed definitions and references describing these phenotypes are presented in Supplementary Table 6. Briefly, phenotype categories analyzed in this study were: stress physiology (hypothalamic–pituitary–adrenal function) (73, 79), 24 h heart rate via telemetry and heart rate variability (HRV) (73), blood pressure (73), morphometrics (42), insulin resistance (42), ovarian functioning (93), and coronary artery atherosclerosis (81). We measured cortisol levels for acute and ACTH challenge tests using RIA kits from DiaSource (IBL America) (73, 79). Electrocardiograms (ECG) were recorded over a 24 h period at 12 and 29 months of experimental diet consumption using a portable ECG telemetry unit (Life Sensing Instrument Co, Tullahoma, TN, United States) (73, 79, 81, 94). Dependent variables derived from the ECG included HRV in 2-h daytime (1600–1800) and nighttime (0100–0300) blocks derived using Nevrokard-HRV software (Nevrokard Kiauta, d. o. o. Izola Slovenia) (73, 95, 96). Blood pressure was measured using high definition oscillometry via tail cuff (73). Ovarian function was assessed during months 16–27 of experimental phase by measuring peak luteal-phase progesterone concentration (ng/ml), cycle length (days), and cycle regularity for each menstrual cycle. Blood sampling was performed 3X a week or progesterone assay while vaginal swabbing was conducted 6X a week for menses (93, 97). Progesterone was determined using a commercially available radioimmunoassay kit (DiaSource Progesterone RIA-CT KIP1458, Louvain-la-Neuve, Belgium). Plasma estradiol was assessed at the time of necropsy using a sensitive estradiol ELISA kit (Cat # IB78239) from IBL America (Minneapolis, MN, United States). Detailed definition and references describing the collected phenotypes are presented in Supplementary Table 6. Relationships between the phenotypes and the normalized transcript levels of the top differentially expressed genes (FDR < 0.05) were analyzed in iliac arteries as a function of diet, and in carotid arteries as a function of social status (relative rank score). To measure correlation coefficient and significance, we conducted a Spearman’s correlation test and adjusted for diet in iliac arteries, and for relative rank in carotid arteries, using the *corr.test* function in R.

## 3 Results

### 3.1 Western diet promoted intimal lesion formation in the left anterior descending coronary arteries

**Figure 1:**
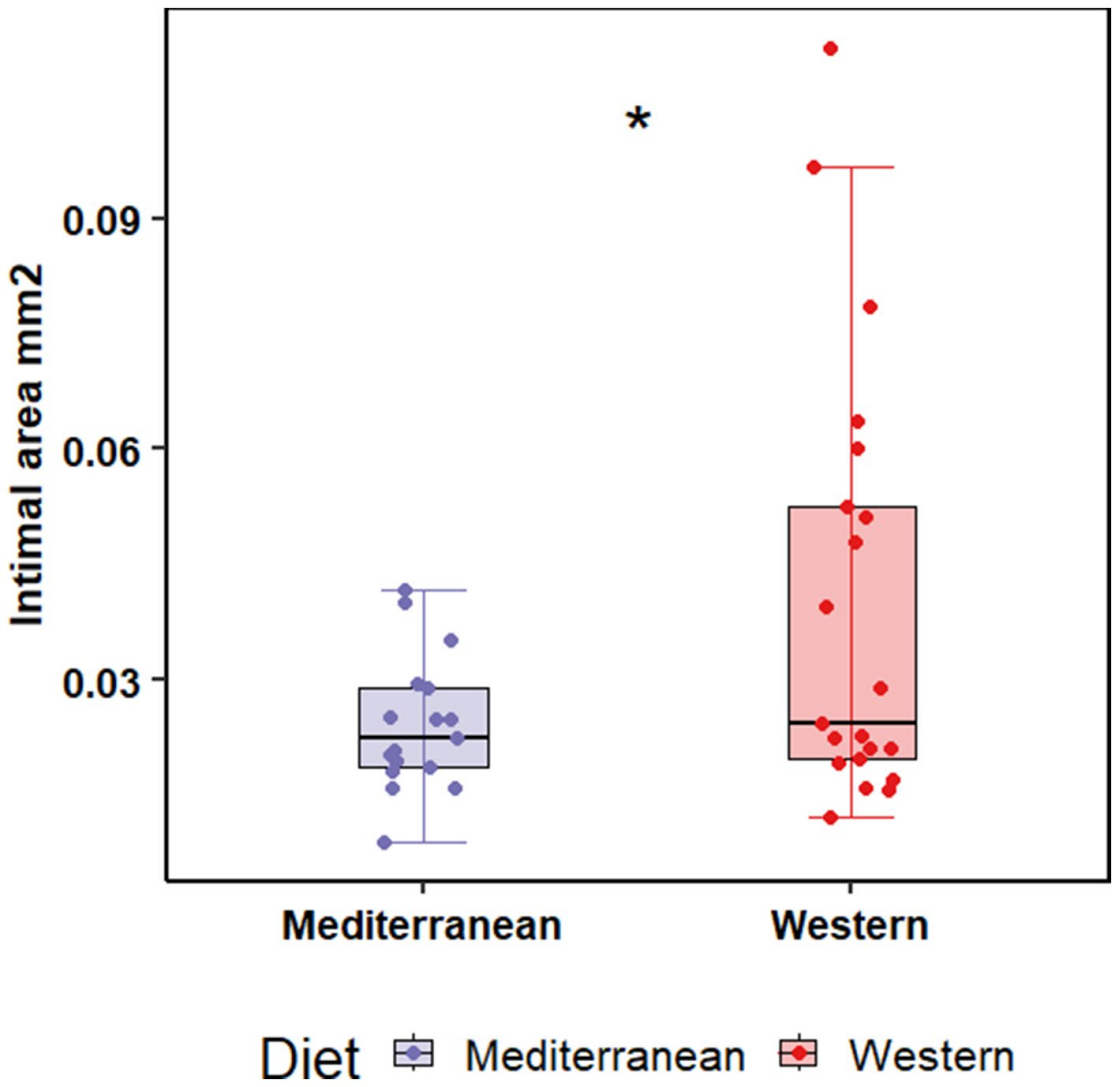
Western diet promoted intimal lesion formation in the proximal left anterior descending coronary compared to the Mediterranean diet. Tissue sections were stained with Verhoeff-van Gieson’s stain, and cross-sectional area of the arterial intima was measured. Two-way ANOVA showed a significant main effect of diet (Western 0.04 ± 0.03 mm^2^ vs. Mediterranean 0.02 ± 0.008 mm^2^; F_(1, 36)_ = 5.25, *p* = 0.03).

Atherosclerosis was assessed in the left anterior descending artery (LAD), left circumflex artery (LCX), right coronary artery (RCA), in the common iliac artery (LCI), and left carotid artery (LCC) as previously described (49). Intimal lesion areas were assessed for the main effects of diet (Western vs. Mediterranean) and social status (dominant vs. subordinate). We also tested the interaction between diet and social status. Western diet fed monkeys had significantly larger intimal lesions in the LAD coronary artery (0.04 ± 0.03 mm^2^; *n* = 21) compared to monkeys fed the Mediterranean diet (0.02 ± 0.008 mm^2^; *n* = 17; F _(1, 36)_ = 5.25, *p* = 0.03; Table 1; Figure 1). There were no significant main effects of diet, social status, or diet by status interactions on lesions in any other arteries.

**Table 1:** Effects of diet and social status on atherosclerosis.

Effect	n	Mean (mm^2^)	Std. Dev. (mm^2^)	Two-way ANOVA			
				Mean Squares	F-statistic	*p*-value	DF
Proximal left anterior descending coronary artery (LAD)							
Diet (West, Med)	21, 17	0.04, 0.02	0.03, 0.008	0.0024	5.25	**0.03**	1,36
Social Status (Sub, Dom)	17, 21	0.03, 0.04	0.017, 0.026	0.0013	2.83	0.1	1,36
Diet x Status	38			0.0006	1.38	0.25	3,34
Proximal left circumflex coronary artery (LCX)							
Diet (West, Med)	21, 17	0.025, 0.024	0.014, 0.013	0.00002	0.11	0.7	1,36
Social Status (Sub, Dom)	17, 21	0.019, 0.028	0.008, 0.016	0.0006	4	**0.05**	1,36
Diet x Status	38			0.0003	2	0.16	3,34
Proximal right coronary artery (RCA)							
Diet (West, Med)	21, 17	0.019, 0.018	0.009, 0.010	0.000002	0.017	0.9	1,36
Social Status (Sub, Dom)	17, 21	0.017, 0.020	0.009, 0.010	0.0001	1.21	0.3	1,36
Diet x Status	38			0.00002	0.23	0.6	3,34
Proximal left common iliac artery (LCI)							
Diet (West, Med)	21, 17	5.1, 4.9	0.22, 0.24	0.15	2.6	0.1	1,36
Social Status (Sub, Dom)	17, 21	5.0, 5.0	0.22, 0.25	0.02	0.4	0.5	1,36
Diet x Status	38			0.06	1.1	0.3	3,34
Proximal left carotid artery (LCC)							
Diet (West, Med)	21, 17	1.6, 1.5	1.7, 1.6	0.1	0.04	0.8	1,36
Social Status (Sub, Dom)	17, 21	1.0, 4.6	1.4, 1.7	9.5	3.7	0.06	1,36
Diet x Status	38			1.1	0.4	0.5	3,34

Mean intimal lesion area (mm2) in collected arteries is measured for each study group, (Western cohort vs. Mediterranean cohort, and subordinate monkeys vs. dominant monkeys). Two-way ANOVA was used to analyze the effect size of diet, social status (dichotomous, dominant vs. subordinate), and the interaction of both on intimal lesion area in each artery. The model used for analysis: Y = *μ* + αdiet + βstatus + (αβ) diet x status + *ϵ*. Bold values are significant @ *p* < 0.05.

### 3.2 Overview of diet and social status effects on the transcriptional profiles in iliac and carotid arteries

Diet was associated with significant differences in transcript levels in iliac but not carotid arteries; while social status was associated with significant differences in transcript levels mainly in the carotid artery, and to a smaller extent in the iliac artery. The social status effect on transcript levels was assessed using both a continuous (relative rank score) and dichotomous (dominant rank vs. subordinate rank) approach; effect size on transcript levels was larger when using the continuous value, thus we are only reporting differential expression in response to the relative rank score in this paper. There was no significant interaction between diet and social status in either artery. A summary of the findings is shown in Table 2.

**Table 2:** Summary of diet and social status differential effects on iliac and carotid arterial transcriptome.

Iliac arteries		Carotid arteries	
Total samples = 35 Western *n = 20*, Mediterranean *n = 15* Dominant *n = 20*, Subordinate *n = 15*		Total samples = 38 Western *n = 21*, Mediterranean *n = 17* Dominant n = 21, Subordinate *n = 17*	
Diet effect	Social status effect	Diet effect	Social status effect
Total genes: 13,020	Total genes: 13,020	Total genes: 13,206	Total genes: 13,206
20 genes (FDR < 0.05)	2 genes (FDR < 0.05)	0 genes (FDR < 0.05)	18 genes (FDR < 0.05)
37 genes (FDR < 0.1)	13 genes (FDR < 0.1)	3 genes (FDR < 0.1)	68 genes (FDR < 0.1)
174 genes (FDR < 0.2)	39 genes (FDR < 0.2)	35 genes (FDR < 0.2)	216 genes (FDR < 0.2)
Compared to the Mediterranean cohort; Down regulated genes in the Western cohort: *PIK3R1, PABPC1, PAQR8, ZNF667, FGGY, EIF4B, ALDH3A2, ANP32A, KDM3B, XPO7, RPS20, TOMM20, and CHCHD7* (FDR < 0.05). Up regulated genes in the Western cohort: *WDR62, PKDCC, SLC29A2, MARS1, RAD21L1, MAMDC4, and ENSMFAG00000052859 [uncharacterized coding protein]* (FDR < 0.05).	Compared to the Dominant monkeys; Down regulated genes in the Subordinate monkeys: *ENSMFAG00000064154 [LncRNA],* and *ENSMFAG00000057515 [small nucleolar RNA U13]* (FDR < 0.05).	Compared to the Mediterranean cohort; Down regulated genes in the Western cohort: *SIRT6, AMOTL2,* and *YIF1A* (FDR < 0.1).	Compared to the Dominant monkeys; Down regulated genes in the Subordinate monkeys: *IRAK1BP1, KIAA0513, SMIM15, PSMD14, TOPORS, ARPC2, and ENSMFAG00000050714 [LncRNA]* (FDR < 0.05). Up regulated genes in the Subordinate monkeys: *KCNQ4, STIM1, TNKS1BP1, CSNK1D, INPPL1, PNPLA7, F10, RAD9A, KCNIP3, ENSMFAG00000059809 [LncRNA], and ENSMFAG00000053865 [secreted protein A0A7N9CS45]* (FDR < 0.05).

#### 3.2.1 Effects of diet and social status on the iliac artery transcriptome

**Figure 2:**
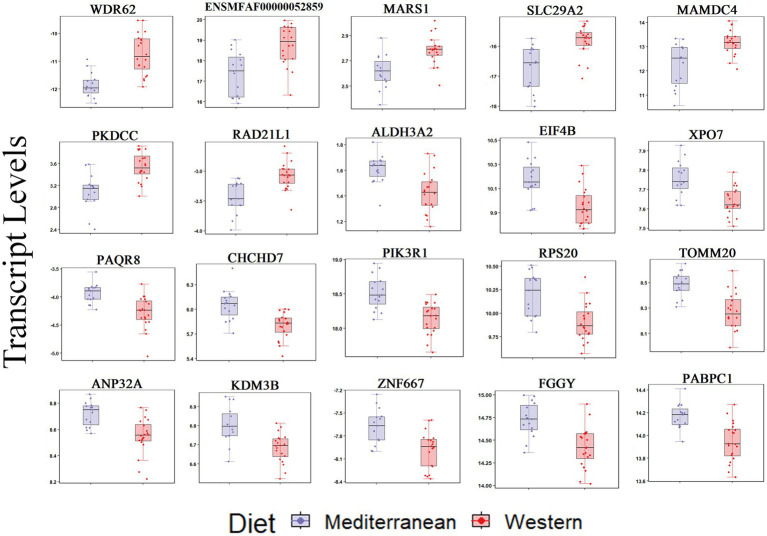
Transcript levels (TMM normalized) differentially expressed by diet in the iliac artery (FDR < 0.05).

For the iliac artery, 20 transcripts were differentially expressed between the two diets (FDR < 0.05; Figure 2). In the Western diet cohort, seven genes were upregulated (*WDR62, PKDCC, SLC29A2, MARS1, RAD21L1, MAMDC4*, and ENSMFAG00000052859 [uncharacterized coding protein]), and 13 genes were downregulated (*PIK3R1, PABPC1, PAQR8, ZNF667, FGGY, EIF4B, ALDH3A2, ANP32A, KDM3B, XPO7, RPS20, TOMM20*, and *CHCHD7*) relative to the Mediterranean diet cohort (Figure 2; Supplementary Table 2).

**Figure 3:**
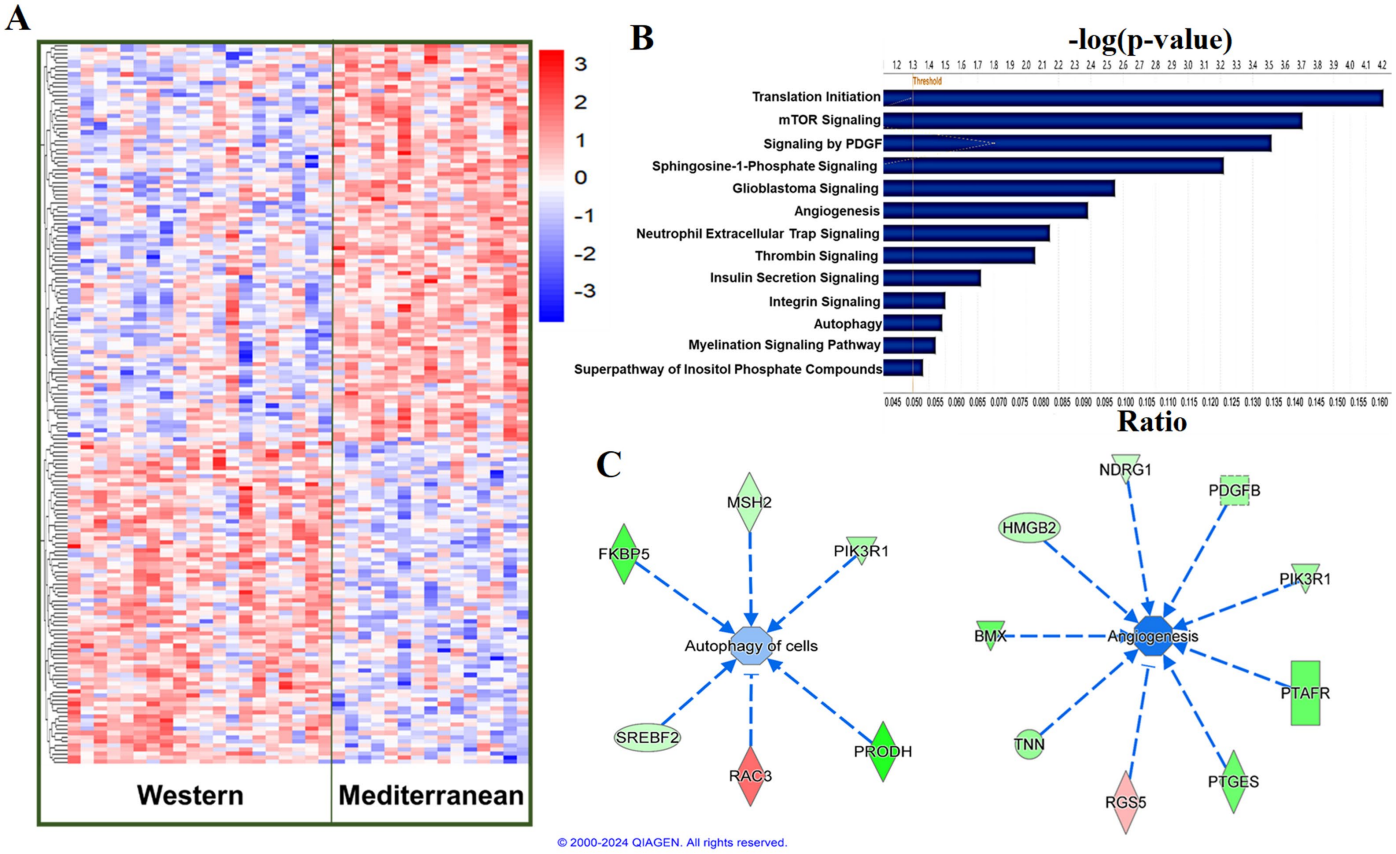
Diet was associated with differential transcriptional profiles in iliac arteries. **(A)** Hierarchal clustering of normalized transcript levels of the top DETs (*n* = 174, FDR < 0.2) in response to diet. Calculated residual transcript level values were converted into z-scores before clustering. **(B)** Canonical pathways identified by Ingenuity Pathway Analysis from 174 DETs with FDRs < 0.2 in iliac arteries. Ratio indicates the number of target genes in the dataset divided by the total number of genes in the pathway. **(C)** Predicted activity of cardiovascular function- related pathways in response to the expression state of DETs (FDR < 0.2) using IPA. Arrows indicate directional relationships. Orange indicates predicted activation and blue indicates predicted inhibition of the biological pathway based on the expression levels of the shown genes in the Western cohort relative to the Mediterranean cohort. Red indicates upregulation and green indicates downregulation of the labeled gene in the Western cohort relative to the Mediterranean cohort.

Using a relaxed FDR threshold of 0.2 to identify a larger set of genes for pathway analysis, we identified 174 diet-associated DETs (Figure 3A; Supplementary Table 2). Regardless of the effect size direction (increased or decreased in Western diet compared to Mediterranean diet), these DETs were enriched for a number of pathways including (1) Translation Initiation, (2) mTOR Signaling, (3) Signaling by PDGF, (4) Autophagy, (5) Thrombin Signaling, (6) Insulin Secretion Signaling Pathway, (7) Integrin Signaling, (8) Neutrophil Extracellular Trap Signaling Pathway (Fisher’s Exact Test, *p* < 0.05; Figure 3B). A complete list of the enriched biological pathways is available in Supplementary Table 3. We also explored pathway enrichment taking into account the effect size direction (increased or decreased in Western diet compared to Mediterranean diet) of the analyzed DETs. We identified and predicted the activity of cardiovascular disease- associated causal networks using IPA, including angiogenesis and autophagy. Autophagy was predicted to be inhibited in the Western diet cohort relative to the Mediterranean cohort (Z-score = −0.572, FDR = 0.02) according to the effect size of the following genes: *FKBP5, MAP1LC3A, MSH2, NDRG1, PIK3R1, PLK1, PRODH, RAC3*, *SREBF2,* and *CAMK1* (Figure 3C). Angiogenesis was also predicted to be inhibited in the Western cohort relative to the Mediterranean diet cohort (Z-score = −1.732, FDR = 0.05) according to the effect size of the following genes: *PALD1, PLK1, RIPK3, SYNE2, AMIGO2*, *BMX, HMGB2, NDRG1, PDGFB, PIK3R1, PTAFR, PTGES,* and *RGS5* (Figure 3C).

Social status was significantly associated with the expression of two genes (FDR < 0.05), both of which are non-coding: (ENSMFAG00000064154 [LncRNA], and ENSMFAG00000057515 [small nucleolar RNA U13]; Table 2). At a relaxed FDR threshold of 0.2, we only identified 39 DETs, which is not enough to conduct a pathway analysis.

#### 3.2.2 Effects of social status on the carotid artery transcriptome

**Figure 4:**
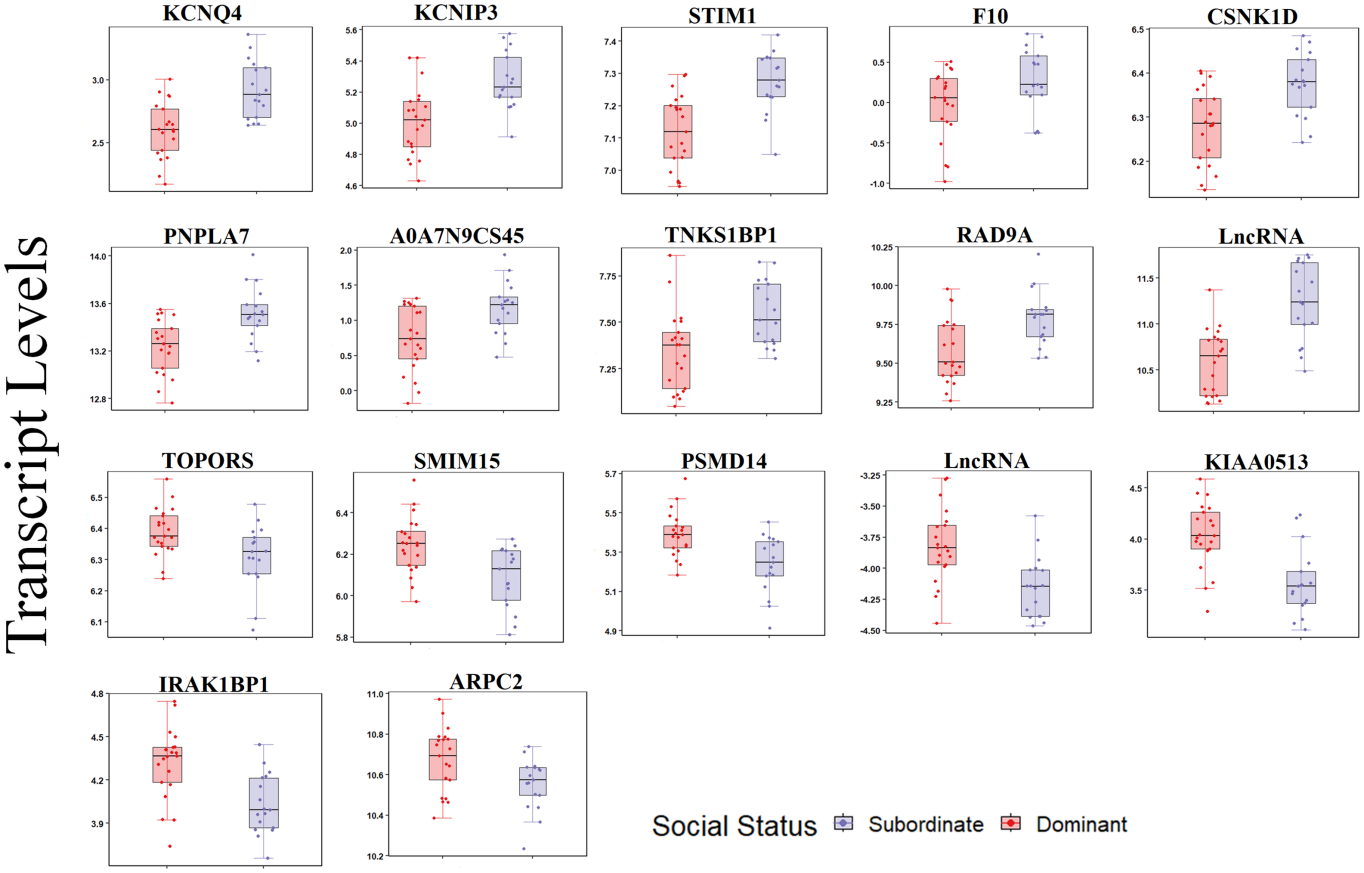
Transcript levels (TMM normalized) differentially expressed by social status in the carotid artery (FDR < 0.05).

For the carotid artery, 18 transcripts were differentially expressed between dominants and subordinates (FDR < 0.05; Figure 4). In the subordinate monkeys, 11 transcripts were upregulated (*KCNQ4, STIM1, TNKS1BP1, CSNK1D, INPPL1, PNPLA7, F10, RAD9A*, *KCNIP3*, ENSMFAG00000059809 [LncRNA], and ENSMFAG00000053865 [secreted protein A0A7N9CS45]), and seven transcripts were downregulated (*IRAK1BP1, KIAA0513, SMIM15, PSMD14, TOPORS, ARPC2*, and ENSMFAG00000050714 [LncRNA]) relative to the dominant monkeys (Figure 4; Supplementary Table 4).

**Figure 5:**
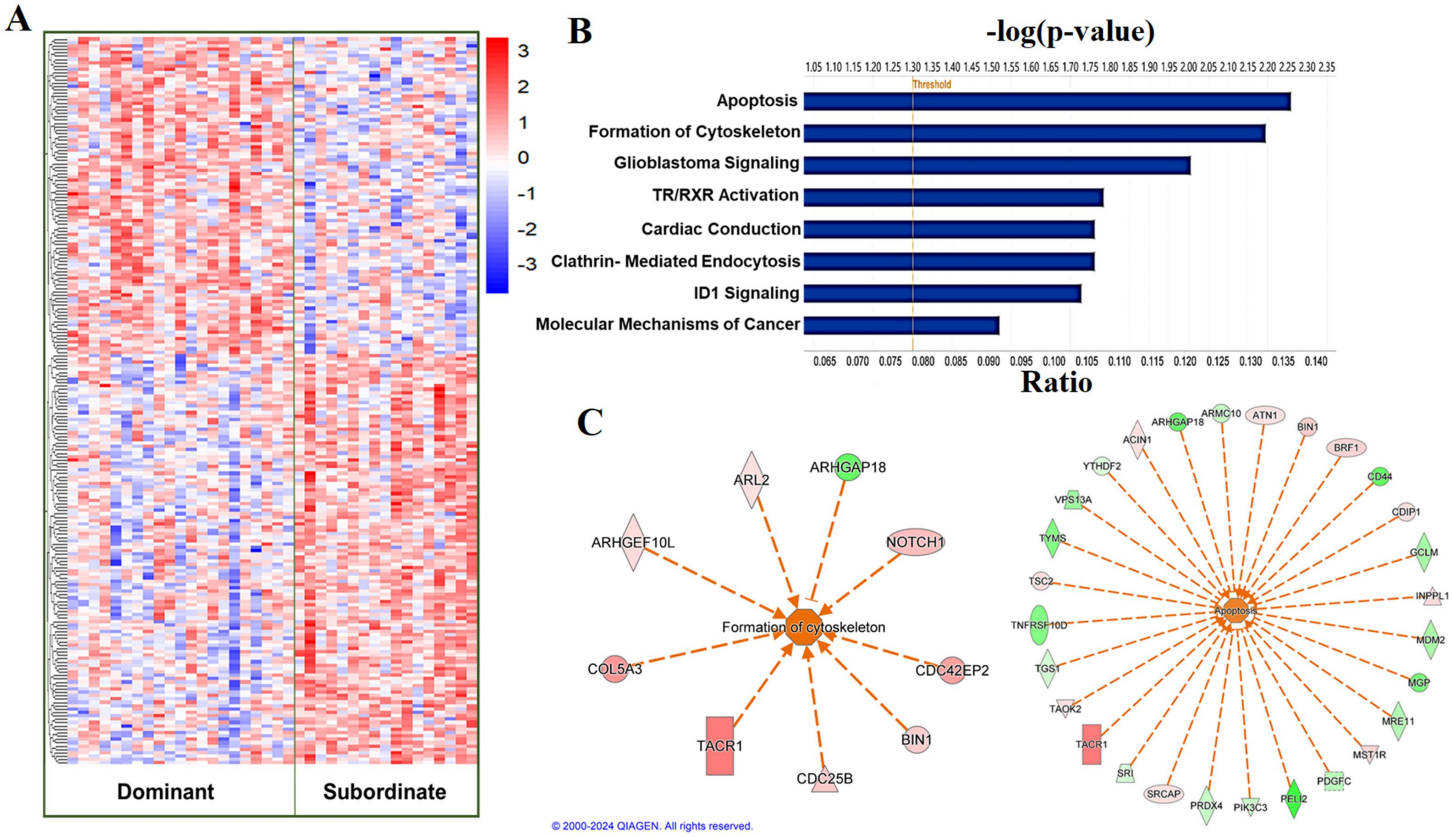
Social status is associated with a differential transcriptional profile in carotid arteries. **(A)** Hierarchal clustering of normalized transcript levels of the top DETs (*n* = 216, FDR < 0.2) in response to social status. Calculated residual transcript level values were converted into z-scores before clustering. **(B)** Canonical pathways identified by Ingenuity Pathway Analysis from 216 DETs with FDRs < 0.2 in carotid arteries. Ratio indicates the number of target genes in the dataset divided by the total number of genes in the pathway. **(C)** Predicted activity of cardiovascular function- related pathways in response to the expression state of DETs (FDR < 0.02) using IPA. Arrows indicate directional relationships. Orange indicates predicted activation and blue indicates predicted inhibition of the biological pathway based on the expression levels of the shown genes in the subordinate cohort relative to the dominant cohort. Red indicates upregulation and green indicates downregulation of the labeled gene in the subordinate cohort relative to the dominant cohort.

At a relaxed FDR threshold of 0.2, we identified 216 social status-associated DETs (Figure 5A; Supplementary Table 4). Regardless of the effect size direction (increased or decreased in subordinates compared to dominants), these DETs were enriched for a number of pathways including (1) Cardiac conduction, (2) ID1 Signaling Pathway, (3) Salvage Pathways of Pyrimidine Ribonucleotides, (4) Glioblastoma Multiforme Signaling, (5) Clathrin-mediated endocytosis, (6) TR/RXR Activation (Fisher’s Exact Test, *p* < 0.05; Figure 5B). A complete list of the enriched biological pathways is available in Supplementary Table 5. We also explored pathway enrichment taking into account the effect size direction (increased or decreased in subordinates compared to dominants) of the analyzed DETs. We identified and predicted the activity of cardiovascular disease- associated causal networks using IPA, including apoptosis and cytoskeleton formation. Cytoskeleton formation was predicted to be activated in subordinate compared to dominant monkeys (Z-score = 2.14, FDR = 0.03) according to the effect size of the following genes: *ARHGAP18, ARHGEF10L, ARL2, CDC25B, CDC42EP2, COL5A3, NOTCH1, BIN1*, and *TACR1* (Figure 5C). Apoptosis was predicted to be activated in subordinate compared to dominant monkeys (Z-score = 1.4, FDR = 0.06) according to the effect size of the following genes: *ACIN1, ARHGAP18, ARMC10, ATN1, BIN1, BRF1, CD44, CDIP1, GCLM, INPPL1, MDM2, MGP, MRE11, MST1R, PDGFC, PELI2, PIK3C3, PRDX4, SRCAP, SRI, TACR1, TAOK2, TGS1, TNFRSF10D, TSC2, TYMS, VPS13A*, and *YTHDF2* (Figure 5C).

There was no main effect of diet on the carotid artery transcriptome (all FDR > 0.05, Table 2). With a relaxed FDR threshold of 0.2, we identified 35 diet dependent transcripts in carotid arteries (Table 2).

### 3.3 Associations of iliac and carotid arteries transcriptomes with physical, behavioral, and physiological phenotypes

Differential transcriptional profiles in iliac and carotid arteries were compared with physical, behavioral, and physiologic phenotypes collected throughout the study using Spearman’s correlation test. A complete list of the analyzed phenotypes and the correlation coefficients with DETs is shown in Supplementary Tables 7, 8.

**Figure 6:**
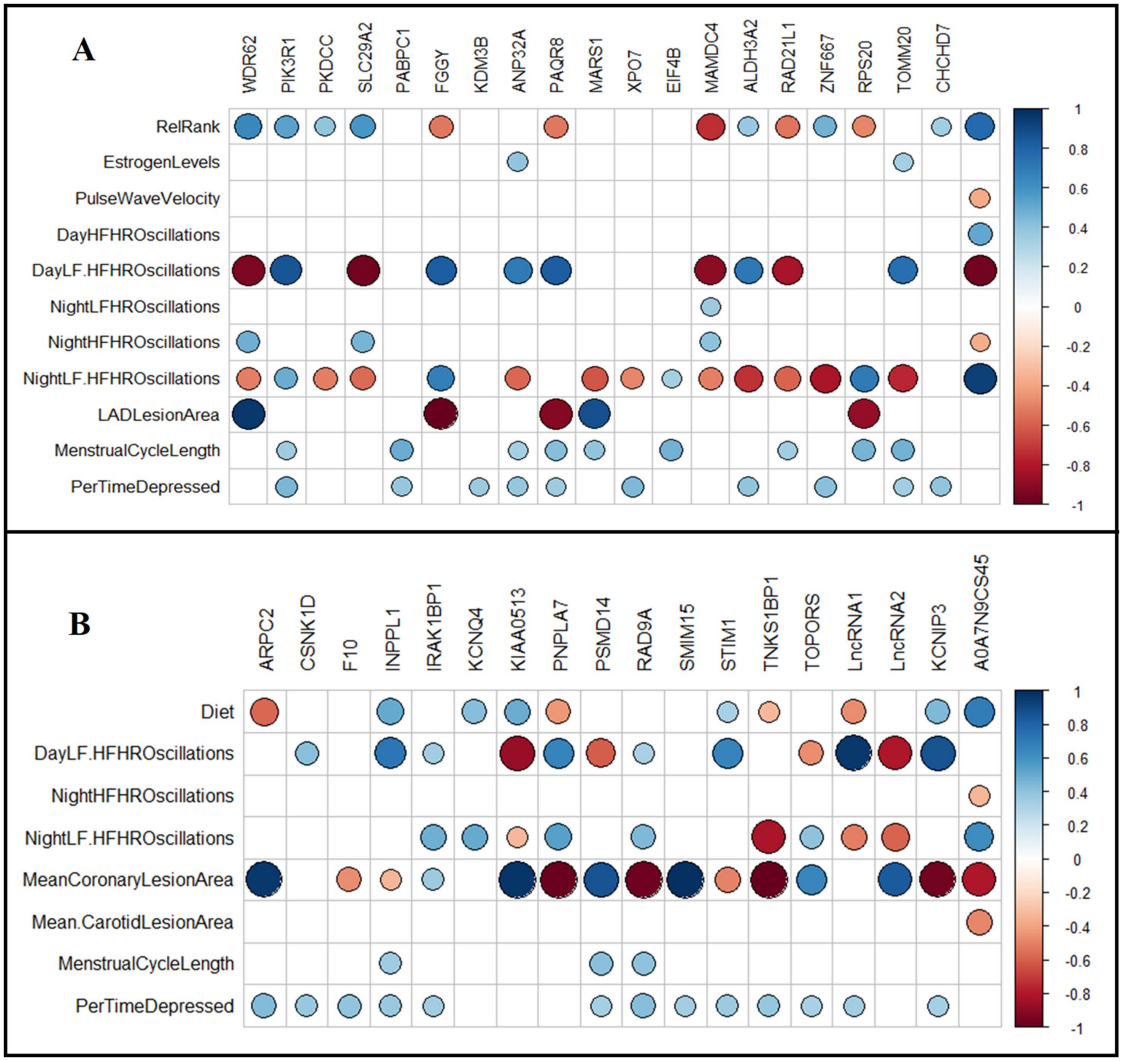
Association of normalized transcript levels of differentially expressed genes in iliac and carotid arteries with physical, behavioral, and physiological phenotypes. **(A)** Diet adjusted Spearman’s correlation between DETs (FDR < 0.05) in iliac arteries with collected phenotypes. **(B)** Relative rank adjusted Spearman’s correlation between DETs (FDR < 0.05) in carotid arteries with collected phenotypes. Spearman’s test was conducted to identify significant correlations. Squares with colored circles inside indicate significant correlations (FDR < 0.05) between the indicated gene and the respective phenotype. Blue indicates positive correlation and red indicates negative correlation. RelRank: continuous value of social status, EstrogenLevels: plasma estrogen levels in pg./mL measured at necropsy, PulseWaveVelocity: pulse wave velocity measured by tail high definition oscillometry, DayHFHROscilation: percentage of high frequency (HF) oscillations during the day at 1600–1800 h, DayLF. HFHROscilation: ratio of LF/HF oscillations during the day at 1600–1800 h, NightLF. HFHROscilation: ratio of LF/HF oscillations during the night at 0100–0300 h, NightLFHROscilation: percentage of low frequency (LF) oscillations during the night at 0100–0300 h, NightHFHROscilation: percentage of high frequency (HF) oscillations during the night at 0100–0300 h, MeanCoronaryLesionArea: mean lesion area of the three coronary arteries collected, LADLesionArea: lesion area of the left anterior descending coronary artery, MeanCarotidLesionArea: mean lesion area of the three left common carotid arteries collected, MenstrualCycleLength: average length of cycles during experimental phase, measured in days, PerTimeDepressed: percent of time spent in depressive behavior.

**Iliac arteries**: Relationships between the top diet associated DETs (FDR < 0.05) and multisystem phenotypes were assessed using Spearman’s test (Figure 6A). Coronary artery atherosclerosis (LAD-lesion area) was positively correlated with *WDR62* and *MARS1* transcript levels, and negatively correlated with *FGGY, PAQR8*, and *RPS20* transcript levels (*p* < 0.05; Figure 6A). Telemetry heart rate variability measures were significantly correlated with the transcript levels of most of the analyzed DETs (*p* < 0.05; Figure 6A). Relative rank was positively correlated with *WDR62, PIK3R1, PKDCC, ALDH3A2, SLC29A2*, *ZNF667,* and *CHCHD7* transcript levels; and negatively correlated with *FGGY, PAQR8, MAMDC4, RAD21L1*, and *RPS20* transcript levels (*p* < 0.05; Figure 6A). The percentage of time spent depressed was positively correlated with *PIK3R1, PABPC1, KDM3B, ANP32A, PAQR8, XPO7, ALDH3A2, TOMM20, CHCHD7,* and *ZNF667* transcript levels (*p* < 0.05; Figure 6A). Menstrual cycle length was positively correlated with *PIK3R1, PABPC1, ANP32A, EIF4B, PAQR8, MARS1, RAD21L1*, *TOMM20, CHCHD7,* and *RPS20* transcript levels (*p* < 0.05; Figure 6A). Estradiol concentration at time of necropsy positively correlated with *ANP32A* and *TOMM20* transcript levels (*p* < 0.05; Figure 6A).

**Carotid arteries**: Relationships between the top status-associated DETs (FDR < 0.05) with multisystem phenotypes were assessed using Spearman’s test (Figure 6B). LAD-lesion area (mm^2^) was positively correlated with *ARPC2, IRAK1BP1, KIAA0513, PSMD14, SMIM15*, and *TOPORS* transcript levels; and negatively correlated with *CSNK1D, KCNQ4, PNPLA7, RAD9A, STM1*, and *TNKS1BP1* transcript levels (*p* < 0.05; Figure 6B). Telemetry heart rate variability measures were correlated with the transcript levels of many DETs (*p* < 0.05; Figure 6B). Diet was positively correlated with *INPPL1, KCNQ4, STIM1, KCNIP3*, and *KIAA0513* transcript levels; and negatively correlated with *ARPC2, PNPLA7,* and *TNKS1BP1* transcript levels (*p* < 0.05; Figure 6B). Percentage of time spent depressed was positively correlated with *ARPC2, CSNK1D, F10, INPPL1, IRAK1BP1, PSMD14, RAD9A, SMIM15, STIM1, TNKS1BP1*, *KCNIP3*, and *TOPORS* transcript levels (*p* < 0.05; Figure 6B). Menstrual cycle length was positively correlated with *INPPL1, PSMD14*, and *RAD9A* transcript levels (*p* < 0.05; Figure 6B).

## 4 Discussion

Our results demonstrate the associations of environmental influences (e.g., diet and social status) with atherosclerosis and vascular biology. Western diet promoted coronary artery atherogenesis and altered the arterial transcriptome in iliac but not carotid arteries relative to the Mediterranean diet. Social status was associated with 18 DETs in the carotid artery transcriptome and two DETs in the iliac artery. Transcriptional profiles in iliac and carotid arteries were significantly correlated with multiple physical, behavioral, and physiological phenotypes collected throughout the study, including coronary atherosclerosis, heart rate variability measures, menstrual cycle length, and depression behavior.

Atherosclerotic lesion development was increased in response to Western diet in the LAD coronary artery but not in any other coronary or peripheral arteries. Endothelial cell biology plays a key role in the initiation, development, and composition of atherosclerotic plaques (98, 99), and both local environmental factors and vascular bed origin influence atherosclerotic plaque development (100). Atherosclerosis susceptibility and lesion characteristics vary across different arterial sites (98, 101) with plaque formation more prevalent in coronary compared to femoral and carotid arteries (99). The LAD is the largest coronary artery, and supplies blood to the left side of the heart (102, 103), and is more atherosclerosis prone than the RCA and LCX (104), as observed in the present study. Intimal lesion area was measured in the sections close to branch points and bifurcations of the arteries; which are atherosclerosis-prone regions with disturbed blood flow promoting endothelial cell dysfunction (105, 106).

Atherosclerosis development was modest in the present study. Female macaques such as those in the present study are relatively protected from atherosclerosis, similar to the protection observed in premenopausal women (107). The Western and Mediterranean diets both contained a cholesterol content of 320 mg / 2000 calories/day with 30% of calories from fat, although the sources and types of fat in the two diets were significantly different, demonstrating that the sources of macronutrients and micronutrients contribute significantly to cardiovascular disease risk.

In the iliac arteries, diet-altered DETs had links to endothelial dysfunction, smooth muscle proliferation and migration, angiogenesis, and abnormal ECM dynamics (108–118). WDR62 (WD repeat-containing protein 62) is involved in the regulation of the cell cycle pathway and interacts directly with other cell cycle proteins to induce spindle formation and mitotic progression (109), and is a regulator of the cardiac myocyte cell cycle and a susceptibility gene for congenital heart defects (CHD) (109, 110). *WDR62* upregulation in the Western cohort may be associated with hyperactive cell cycle and increased proliferation. *WDR62* iliac transcripts were strongly positively correlated with plaque formation in the LAD coronary artery.

*PIK3R1* (Phosphoinositide-3-Kinase Regulatory Subunit 1) mRNA expression was lower in the Western diet cohort relative to the Mediterranean diet cohort. PIK3R1 is involved in the regulation of insulin and PIK3R1/AKT pathways and exerts anti-apoptotic and protective effects on endothelial cells. *PIK3R1* downregulation and/or inhibition is shown to promote atherosclerosis (112–115). Western diet also downregulated *ANP32A* (Acidic Nuclear Phosphoprotein 32 Family Member A), a translational repressor of the Wnt pathway known to impact cardiac disease (108). *ANP32a*-deficient mice have activated Wnt signaling and develop cardiac hypertrophy (108). The transcription factor ZNF667 (Zinc Finger Protein 667), can repress the expression of anti-angiogenesis genes and regulate expression of Wnt signaling genes in vascular endothelial cells, which in turn promote angiogenesis pathway (111). *ZNF667* transcripts were downregulated in the Western diet cohort relative to the Mediterranean diet cohort, suggesting reduced angiogenesis.

Western diet downregulated transcripts for *FGGY* (FGGY carbohydrate kinase domain containing), and *PAQR8* (Progestin and AdipoQ Receptor Family Member 8) a transmembrane progesterone receptor involved in steroid hormone response pathways. Both genes have been associated with vascular disease, cardiac structure and function, and ischemic stroke (116–118). Our analysis showed that transcript levels of both genes were strongly negatively correlated with intimal area in the LAD coronary artery, suggesting an association with plaque formation.

Ingenuity pathway analysis of the significant DETs in iliac arteries predicted an inhibition of autophagy in the Western diet cohort relative to the Mediterranean diet cohort. Autophagy is responsible for the degradation of proteins and organelles, to maintain metabolic homeostasis within the cell (119). Autophagy dysfunction is closely associated with many aging-related diseases including cardiovascular disorders (120). Multiple studies have demonstrated a protective effect of maintaining basal autophagy in atherosclerosis (121–123). Impaired autophagy leads to accumulation of cytotoxic and dysfunctional organelles and aggregates (124), contributing to endothelial dysfunction, monocyte/macrophage migration and adhesion that can lead to the development of atherosclerosis (125).

Collectively, this present study demonstrates that diet had a significant impact on coronary and iliac arteries that are associated with promoting atherosclerosis. In addition to this data, we have also shown before that diet induced numerous other effects spanning several tissues and phenotypes. Our previous findings demonstrated that compared to a Western diet, Mediterranean diet was protective against neuroinflammation (126) and pro-inflammatory monocyte polarization (127), reduced triglyceride levels (42), reduced social isolation and anxiety (67), protected against ovarian dysfunction (93) and insulin resistance ^19^, and enhanced stress resilience as indicated by lower sympathetic activity, overt heart rate responses to acute stress, and lower cortisol responses to acute psychological stress (73).

These findings highlight the multifaceted consequences of dietary composition on a wide range of physiological systems, suggesting that nutritional habits impact multiple health outcomes including cardiovascular, metabolic, endocrine, neurological, and behavioral endpoints, which shows the complexity of diet effects. This demonstrates the strength of this randomized preclinical approach in which it allows for comprehensive assessment of these widespread effects, providing strong evidence for the role of diet as a critical modulator of health and disease.

Diet did not induce major effects on the transcriptional profile of the carotid artery; on the other hand, social status- a proxy for psychosocial stress – impacted patterns of gene expression linked to dysregulated vascular tone and smooth muscle contractility, apoptosis, endothelial dysfunction, and abnormal ECM dynamics (128–132). Upregulation of *KCNQ4* (voltage-gated potassium channel subunit K_v_7.4) and *KCNIP3* (potassium voltage-gated channel interacting protein 3) observed in the subordinates relative to dominants, has been shown previously in the literature to be associated with dysregulated vascular tone and contractility of carotid arteries. KCNQ4 forms a potassium channel that enables the selective passage of potassium ions across the cell membrane and play a critical role in the regulation of neuronal excitability, and the cardiac action potential (128). *KCNQ4* is expressed in the central nervous system, as well as carotid, thoracic, and femoral arteries, specifically in the cell membranes of vascular smooth muscle cells (VSMC) (133). Activation of K_v_7.2–5 channels results in vasodilation and relaxation of carotid arteries, while inhibition causes vasoconstriction and contraction, indicating that these channels play a significant role in controlling arterial tone of carotid arteries (128, 133). KCNIP3 plays a role in regulating potassium current across cell membrane and in turn regulates arterial tone. The transcript levels of both *KCNQ4* and *KCNIP3* are significantly correlated with heart rate variability measures, further suggesting an association between *KCNQ4* and *KCNIP3* transcript levels and dysregulated vascular tone in subordinate monkeys.

*STIM1* (stromal interaction molecule 1), upregulated in subordinate carotids, functions as a calcium sensor that mediates Ca^2+^ influx after depletion of intracellular Ca^2+^ stores to regulate VSMC contractility. Increased activity of STIM1 can cause vasoconstriction and may affect the development or progression of hypertension (129, 130). STIM1-regulated Ca^+2^ homeostasis is crucial for induction of VSMC proliferation, and development. *STIM1* mRNA and protein levels were reported to be upregulated *in vivo* in VSMC from balloon-injured rat carotid arteries compared to non-injured control vessels, and the knockdown of *STIM1* suppressed neo-intimal hyperplasia in a rat carotid artery balloon injury model (134–136). STIM1 was also found to be upregulated in human coronary artery VSMCs and major regulator of their proliferation (137). Proliferation and migration of VSMC and ECM rearrangement are important early mechanisms in the pathogenesis of vascular disease.

Subordinate monkeys carotid arteries also showed increased *INPPL1* (inositol polyphosphate phosphatase like1), also called Shc homology 2–containing inositol 5 phosphatase-2 (SHIP2), a lipid phosphatase that inhibits Akt phosphorylation and activation, inhibits insulin signaling, and induces apoptosis (131). Apoptosis induction plays a critical role in the pathogenesis of atherosclerotic cardiovascular disease (138). IPA pathway analysis of DETs predicted apoptosis to be activated in the subordinate monkeys relative to dominant monkeys. SHIP2 was also found to be associated with hypertension, obesity and type 2 diabetes (139). Carotid arteries of subordinate monkeys also showed upregulation of *F10* (coagulation factor X), an important factor in forming blood clots at sites of injuries. Beyond the coagulation cascade, F10 can also affect inflammatory signaling by acting on endothelial, VSMCs and inflammatory cells, again associated with the pathogenesis of atherosclerosis. Coagulation protease inhibitors have been shown to attenuate atherogenesis in animal models ^118^. *PNPLA7* and *CSNK1D* were upregulated in subordinate relative to dominant monkeys; PNPLA7 (patatin-like phospholipase), a regulator of adipocyte differentiation induced by metabolic stimuli, was shown to be upregulated in subjects with hypertension (140). CSNK1D (Casein kinase I delta) regulates cellular growth and survival including Wnt signaling, DNA repair and circadian rhythms. Large scale omics studies have shown that CSNK1D is associated with immune and inflammatory responses, LDL cholesterol concentrations, cardiometabolic risk, and myocardial infarction (141, 142). Subordinate monkey carotid arteries had reduced transcripts for *TOPORS*, an E3 ubiquitin-protein ligase that was shown in the literature to interact with syndecan-1 and inhibit cell growth in murine arterial smooth muscle cells (143).

It is interesting that social subordination had a higher impact on the transcriptional profile of carotid arteries than iliac arteries. Previous work demonstrated elevated carotid artery atherosclerosis in subordinate female monkeys (64). Additionally, Shively et al., showed previously that social subordination in macaques is stressful, evident by the heightened cortisol response to challenges (47, 50, 59), and elevated heart rate and blood pressure (50, 80, 144) which can strain the carotids. Psychosocial stress can also increase inflammation and cholesterol levels, contributing to plaque formation (52, 71). It was also shown that subordinate female long-tail macaques exhibit more depressive behavior than their dominant counterparts (80, 94). These characteristics of subordinates may explain in part the significant social status differences in gene expression of carotid arteries.

Given these data, and what is known previously in literature about SDH, it is critical to address SES-related disparities in vascular health through targeted interventions and policy reforms. This can be done by implementing comprehensive intervention strategies to enhance awareness of social disparities and their influence on disease progression and treatment outcomes. Additionally, the quality of diet of socially disadvantaged people must be improved, by implementing policies aimed at increasing the affordability and accessibility of healthy foods. Educational programs that promote nutrition awareness are critical. These efforts are vital for improving public health outcomes and ensuring equitable access to nutritious foods across all socioeconomic levels. Moreover, integrating social and biological data into clinical decision-making processes and risk assessment models is crucial for developing predictive strategies that incorporate both traditional biomarkers and socio-markers, thereby enhancing precision medicine.

This study provides evidence in support of differential effects of diet and psychosocial stress on vascular health. Strengths of the study include the use of stratified randomization to equalize the treatment groups, standardized and consistent environmental conditions across all participants, and the use of a well-established NHP model of cardiovascular and other health outcomes. Limitations include the use of bulk RNAseq to assess transcriptomic changes in arteries, which are complex tissues with cell types that are diverse in both provenance and function. Thus, we were unable to disentangle the contributions of various cell types, such as smooth muscle cells, fibroblasts, endothelial cells, and immune cell populations to the overall effects of diet and social stress. Future studies utilizing single-cell RNA sequencing of arterial tissues would facilitate a comprehensive analysis of cell specific responses to these environmental stressors. Our trial was also limited by relatively small sample size with limited statistical power to capture diet and social status interactions. This study did not include a group maintained on a standard laboratory chow diet, because “monkey chow” is not similar to any diet that humans or NHP eat. Furthermore, monkey chow has some very specific characteristics which may influence experimental results such as high levels of soy isoflavones which are selective estrogen receptor modulators. Our aim was to model human dietary patterns more realistically by directly comparing Western and Mediterranean diets. Finally, this study sample was focused on females, therefore additional study will be needed to fully understand the effects of diet and psychosocial stress on atherosclerosis and gene expression in male subjects.

In conclusion, our data demonstrates differential diet and social status effects dependent on arterial sites. Diet was associated with differences in coronary atherosclerosis and in the iliac artery transcriptome, while social status was associated with differences in the carotid artery transcriptome with a lesser effect in the iliac. In both iliac and carotid arteries, the resulting dysregulated pathways in response to diet and social status are all characteristics of atherosclerosis development and progression. These findings further demonstrate the detrimental effects of Western dietary habits and the stress from social subordination on vascular health. They also point toward the mechanisms through which Mediterranean diets may provide protection from the development of cardiovascular disease. Ongoing analyses are underway to assess relationships between arterial, adipose, and brain responses to diet and social status in this study.

## Data Availability

The original contributions in the study are included in the article/Supplementary material, further inquiries can be directed to the corresponding author.

## Footnotes

^1^ www.qiagen.com/ingenuity

## Glossary

CVD: Cardiovascular Disease
DDS: Dietary Diversity Score
DET: Differentially Expressed Transcripts
DQS: Dietary Quality Score
ECG: Electrocardiogram
ECM: Extracellular Matrix
IPA: Ingenuity Pathway Analysis
LAD: Left Anterior Descending
LCX: Left Circumflex
NHP: Nonhuman Primate
PCA: Principal Component Analysis
RCA: Right Coronary Artery
RIN: RNA Integrity Number
SDH: Social Determinants Of Health
SES: Socioeconomic Status
TMM: Trimmed Mean Of M-Value
WGCNA: Weighted Gene Co-Expression Network Analysis
ACIN1: Apoptotic Chromatin Condensation Inducer 1
ALDH3A2: Aldehyde Dehydrogenase 3 Family Member A2
ANP32A: Acidic Nuclear Phosphoprotein 32 Family Member A
ARHGAP18: Rho Gtpase Activating Protein 18
ARHGEF10L: Rho Guanine Nucleotide Exchange Factor 10 Like
ARL2: ARF Like Gtpase
ARMC10: Armadillo Repeat Containing 10
ARPC2: Actin-Related Protein 2/3 Complex Subunit 2
ATN1: Atrophin 1
BIN1: Bridging Integrator 1
BRF1: BRF1 General Transcription Factor IIIB Subunit
CD44: CD44- Receptor For Hyaluronic Acid
CDC25B: Cell Division Cycle 25
CDC42EP2: CDC42 Effector Protein 2
CDIP1: Cell Death Inducing P53 Target 1
CHCHD7: Coiled-Coil-Helix-Coiled-Coil-Helix Domain Containing 7
COL5A3: Collagen Type V Alpha 3 Chain
CSNK1D: Casein Kinase 1 Delt
EIF4B: Eukaryotic Translation Initiation Factor 4B
ENSMFAG00000050714: [Lncrna]
ENSMFAG00000052859: Unnamed Gene
ENSMFAG00000053865: [Secretedproteina0a7n9cs45]
ENSMFAG00000057515: Secreted Protein A0A7N9CS45
ENSMFAG00000059809: [Lncrna]
ENSMFAG00000064154: [Lncrna]
F10: Coagulation Factor X
FGGY: FGGY Carbohydrate Kinase Domain-Containing Protein
GCLM: Glutamate-Cysteine Ligase Modifier Subunit
INPPL1: Inositol Polyphosphate-5 Phosphatase-Like 1
IRAK1BP1: Interleukin 1 Receptor Associated Kinase 1 Binding Protein 1
KCNIP3: Potassium Voltage-Gated Channel Interacting Protein 3
KCNQ4: Potassium Voltage-Gated Channel Subfamily Q Member 4
KDM3B: Lysine Demethylase 3B
KIAA0513: Undefined Gene
MAMDC4: MAM Domain Containing 4
MARS1: Methionyl-tRNA Synthetase 1
MDM2: Mouse Double Minute 2 Homolog
MGP: Matrix Gla Protein
MRE11: MRE11 Homolog, Double Strand Break Repair Nuclease
MST1R: Macrophage Stimulating 1 Receptor
NOTCH1: Notch Receptor 1
PABPC1: Polyadenylate-Binding Protein 1
PAQR8: Progestin and AdipoQ Receptor Family Member 8
PDGFC: Platelet Derived Growth Factor C
PELI2: Pellino E3 Ubiquitin Protein Ligase Family Member 2
PIK3C3: Phosphatidylinositol 3-Kinase Catalytic Subunit Type 3
PIK3R1: Phosphatidylinositol 3-Kinase
PKDCC: Protein Kinase Domain Containing, Cytoplasmic
PNPLA7: Patatin Like Domain 7, Lysophospholipase
PRDX4: Peroxiredoxin 4
PSMD14: Proteasome 26S Subunit, Non-Atpase 14
RAD21L1: RAD21 Cohesin Complex Component Like 1
RAD9A: RAD9 Checkpoint Clamp Component A
RPS20: Ribosomal Protein S20
SLC29A2: Solute Carrier Family 29 Member 2
SMIM15: Small Integral Membrane Protein 1
SRCAP: Snf2 Related CREBBP Activator Protein
SRI: Sorcin
STIM1: Stromal Interaction Molecule 1
TACR1: Tachykinin Receptor 1
TAOK2: Thousand And One Amino Acid Protein Kinase
TGS1: Trimethylguanosine Synthase 1
TNFRSF10D: TNF Receptor Superfamily Member 10d
TNKS1BP1: Tankyrase 1 Binding Protein 1
TOMM20: Translocase Of Outer Mitochondrial Membrane 20
TOPORS: TOP1 Binding Arginine/Serine Rich Protein, E3 Ubiquitin Ligase
TSC2: TSC Complex Subunit 2 (Tuberin)
TYMS: Thymidylate Synthetase
VPS13A: Vacuolar Protein Sorting 13 Homolog A
WDR62: WD Repeat-Containing Protein 62
XPO7: Exportin 7
YTHDF2: YTH N6-Methyladenosine RNA Binding Protein F2 2
ZNF667: Zinc Finger Protein 667
