# Supplementary material for: Differential effects of Mediterranean vs. Western diets on coronary atherosclerosis and peripheral artery transcriptomics
Source: Front Nutr. 2025 Jul 10;12:1564741. doi: 10.3389/fnut.2025.1564741 (PMC12288352; doi:10.3389/fnut.2025.1564741)
Supplement: Supplementary file 1 [file Table_1.docx]

**Supplementary Tables**

**Supplementary Table 1:** **Experimental Diets Composition**. Composition of experimental *vs.* human diets. Some macronutrient values have been rounded to the nearest whole number.

| **Diet Composition** | **Human** | | **Nonhuman Primates** | | |
| --- | --- | --- | --- | --- | --- |
|  | **WEST** | **MED** | **WEST** | **MED** | **Lab Chow** |
|  | % of Calories | | % of Calories | | |
| **Protein** | 15 | 17 | 16 | 16 | **18** |
| **Carbohydrates** | 51 | 51 | 54 | 54 | **69** |
| **Fat** | 33 | 32 | 31 | 31 | **13** |
|  | % Total Fats | | % Total Fats | | |
| **Saturated** | **33** | 21 | 36 | 21 | **26** |
| **Monounsaturated** | 36 | **56** | 36 | 57 | **28** |
| **Polyunsaturated** | 24 | 15 | 26 | 20 | **32** |
| **w6:w3 Fatty Acids** | **15:01** | **2.1-3:1** | 14.8:1 | 2.9:1 | **12:01** |
| **Cholesterol (mg/cal)** | 0.13 | 0.16 | 0.16 | 0.15 | **trace** |
| **Fiber (g/Cal)** | 0.01 | 0.03 | 0.02 | 0.04 | **0.01** |
| **Sodium (mg/Cal)** | **1.7** | **1.3** | 1.7 | 1 | **0.25** |

**Supplementary Table 2.** **Differentially expressed genes as a function of diet in iliac arteries at an FDR threshold of 0.2**. Beta and "SE" refer to the beta coefficient and standard error, respectively. Bold = FDR < 0.05. Positive beta means upregulation and negative beta means downregulation in the Western-cohort relative to the Mediterranean-cohort. Transcripts are organized from smallest to largest FDR. Bold indicates FDR values <0.05.

| **Gene ID** | **Gene Names** | **beta** | **SE** | **p** | **FDR** |
| --- | --- | --- | --- | --- | --- |
| ENSMFAG00000045599 | **WDR62** | 1.11828945 | 0.045636259 | 1.65E-07 | **0.002** |
| ENSMFAG00000045395 | **PIK3R1** | -0.379613766 | 0.006450063 | 2.28E-06 | **0.012** |
| ENSMFAG00000003478 | **TOMM20** | -0.201847052 | 0.001949386 | 4.84E-06 | **0.013** |
| ENSMFAG00000003585 | **PKDCC** | 0.456909089 | 0.00985157 | 4.16E-06 | **0.013** |
| ENSMFAG00000039334 | **SLC29A2** | 0.927055693 | 0.044628438 | 1.14E-05 | **0.020** |
| ENSMFAG00000044845 | **PABPC1** | -0.22579475 | 0.002632917 | 1.08E-05 | **0.020** |
| ENSMFAG00000040366 | **CHCHD7** | -0.250822078 | 0.003452439 | 1.97E-05 | **0.030** |
| ENSMFAG00000037904 | **FGGY** | -0.303119461 | 0.005264235 | 2.94E-05 | **0.032** |
| ENSMFAG00000042919 | **KDM3B** | -0.118253451 | 0.000857309 | 5.37E-05 | **0.032** |
| ENSMFAG00000043083 | **ANP32A** | -0.172521552 | 0.001744348 | 3.62E-05 | **0.032** |
| ENSMFAG00000004629 | **PAQR8** | -0.342792565 | 0.006916049 | 3.76E-05 | **0.032** |
| ENSMFAG00000010123 | **MARS1** | 0.169933891 | 0.00171986 | 4.17E-05 | **0.032** |
| ENSMFAG00000030886 | **XPO7** | -0.117133433 | 0.000792918 | 3.19E-05 | **0.032** |
| ENSMFAG00000037195 | **EIF4B** | -0.221007292 | 0.002941133 | 4.60E-05 | **0.032** |
| ENSMFAG00000000794 | **MAMDC4** | 0.970819607 | 0.057243104 | 4.96E-05 | **0.032** |
| ENSMFAG00000032076 | **ALDH3A2** | -0.19697144 | 0.002294725 | 3.92E-05 | **0.032** |
| ENSMFAG00000032243 | **RAD21L1** | 0.346473137 | 0.007303826 | 5.03E-05 | **0.032** |
| ENSMFAG00000037010 | **ZNF667** | -0.32338253 | 0.006412073 | 5.38E-05 | **0.032** |
| ENSMFAG00000052859 | **NA** | 1.386784769 | 0.119536854 | 6.04E-05 | **0.034** |
| ENSMFAG00000053482 | **RPS20** | -0.290367801 | 0.005468178 | 8.61E-05 | **0.046** |
| ENSMFAG00000063900 | NA | 1.015942147 | 0.06868402 | 0.0001 | 0.052 |
| ENSMFAG00000002566 | NA | -0.699119244 | 0.032602915 | 0.0001 | 0.052 |
| ENSMFAG00000038475 | WIF1 | -1.42288302 | 0.137494903 | 0.0001 | 0.058 |
| ENSMFAG00000064359 | NA | -1.697251137 | 0.201052427 | 0.0002 | 0.068 |
| ENSMFAG00000035816 | NA | 1.551970063 | 0.16945039 | 0.0002 | 0.070 |
| ENSMFAG00000050142 | NA | 0.741072685 | 0.039782694 | 0.0002 | 0.079 |
| ENSMFAG00000031430 | NA | -0.591536095 | 0.025362958 | 0.0002 | 0.079 |
| ENSMFAG00000031697 | PDGFB | -0.39216689 | 0.011271762 | 0.0002 | 0.079 |
| ENSMFAG00000048901 | DOC2B | -0.829288135 | 0.050161956 | 0.0002 | 0.079 |
| ENSMFAG00000054717 | NA | 0.485463644 | 0.017203701 | 0.0002 | 0.079 |
| ENSMFAG00000040386 | RANGAP1 | 0.197562975 | 0.002878893 | 0.0002 | 0.080 |
| ENSMFAG00000040831 | NA | -0.288645779 | 0.006266678 | 0.0003 | 0.085 |
| ENSMFAG00000043615 | HYAL3 | 0.368546707 | 0.010186561 | 0.0003 | 0.085 |
| ENSMFAG00000001255 | USP35 | 0.301801975 | 0.006863871 | 0.0003 | 0.085 |
| ENSMFAG00000044160 | NTRK3 | 0.27862152 | 0.00592347 | 0.0003 | 0.090 |
| ENSMFAG00000041105 | HSDL1 | -0.210766532 | 0.003411501 | 0.0003 | 0.091 |
| ENSMFAG00000059678 | NA | 0.304452343 | 0.007169572 | 0.0003 | 0.093 |
| ENSMFAG00000002787 | ABCB10 | -0.189275797 | 0.002826367 | 0.0004 | 0.101 |
| ENSMFAG00000034552 | RIPK3 | -0.552165094 | 0.023965422 | 0.0004 | 0.101 |
| ENSMFAG00000002376 | LIMA1 | 0.273866261 | 0.005977881 | 0.0004 | 0.106 |
| ENSMFAG00000038790 | RBBP5 | -0.150224569 | 0.001813306 | 0.0004 | 0.106 |
| ENSMFAG00000038668 | SYNE2 | -0.189714693 | 0.00288666 | 0.0004 | 0.106 |
| ENSMFAG00000032387 | GGA2 | -0.155806089 | 0.001959646 | 0.0004 | 0.107 |
| ENSMFAG00000059613 | DIRAS3 | -0.500588225 | 0.020397533 | 0.0005 | 0.109 |
| ENSMFAG00000031782 | MYG1 | 0.258901157 | 0.005464812 | 0.0005 | 0.109 |
| ENSMFAG00000042257 | MAPKBP1 | 0.21990062 | 0.004011998 | 0.0005 | 0.113 |
| ENSMFAG00000055032 | NA | 0.19836887 | 0.003353241 | 0.0006 | 0.113 |
| ENSMFAG00000001399 | EPS8 | -0.333976447 | 0.009457253 | 0.0006 | 0.113 |
| ENSMFAG00000003467 | FTSJ1 | 0.165544169 | 0.002318501 | 0.0006 | 0.113 |
| ENSMFAG00000030034 | XPNPEP1 | 0.230778997 | 0.004495716 | 0.0006 | 0.113 |
| ENSMFAG00000033682 | THAP12 | -0.210814436 | 0.00377358 | 0.0006 | 0.113 |
| ENSMFAG00000037097 | IDS | -0.194110426 | 0.003216172 | 0.0006 | 0.113 |
| ENSMFAG00000037512 | TMEM198 | 0.437110154 | 0.01612414 | 0.0006 | 0.113 |
| ENSMFAG00000048002 | NA | -0.688487357 | 0.040314979 | 0.0006 | 0.113 |
| ENSMFAG00000000377 | CENPV | 0.242339435 | 0.005019616 | 0.0006 | 0.113 |
| ENSMFAG00000037723 | NA | 0.229663973 | 0.004478351 | 0.0006 | 0.113 |
| ENSMFAG00000041055 | CTNS | 0.225181283 | 0.00433261 | 0.0006 | 0.113 |
| ENSMFAG00000041927 | ZNF653 | 0.298625209 | 0.007420382 | 0.0005 | 0.113 |
| ENSMFAG00000056756 | NA | 0.407875347 | 0.013857793 | 0.0005 | 0.113 |
| ENSMFAG00000033241 | PLOD3 | 0.224306013 | 0.004400816 | 0.0007 | 0.114 |
| ENSMFAG00000034584 | RFT1 | -0.203418846 | 0.003593842 | 0.0007 | 0.114 |
| ENSMFAG00000037036 | TRIM4 | -0.156479675 | 0.002178093 | 0.0008 | 0.114 |
| ENSMFAG00000038086 | FAM161B | -0.248175421 | 0.005408562 | 0.0007 | 0.114 |
| ENSMFAG00000042336 | RGS5 | 0.311897102 | 0.008470818 | 0.0007 | 0.114 |
| ENSMFAG00000051300 | NA | -0.406878629 | 0.014418225 | 0.0007 | 0.114 |
| ENSMFAG00000061843 | NA | -0.353335648 | 0.011005402 | 0.0008 | 0.114 |
| ENSMFAG00000065124 | LDLRAP1 | 0.281690319 | 0.007034466 | 0.0008 | 0.114 |
| ENSMFAG00000004699 | EIF3H | -0.189296299 | 0.003189845 | 0.0008 | 0.114 |
| ENSMFAG00000038458 | C8orf48 | -0.530216871 | 0.024581502 | 0.0007 | 0.114 |
| ENSMFAG00000039573 | PALD1 | -0.448520362 | 0.017936037 | 0.0008 | 0.114 |
| ENSMFAG00000045841 | COL5A2 | 0.266654618 | 0.006304024 | 0.0008 | 0.114 |
| ENSMFAG00000001635 | MSH2 | -0.27757303 | 0.006748557 | 0.0007 | 0.114 |
| ENSMFAG00000003552 | EVI2A | -0.751880337 | 0.049794331 | 0.0008 | 0.114 |
| ENSMFAG00000030946 | ZNF426 | -0.246371286 | 0.005399718 | 0.0008 | 0.114 |
| ENSMFAG00000032435 | GLOD4 | 0.25438267 | 0.005745078 | 0.0008 | 0.114 |
| ENSMFAG00000065510 | NA | 0.281174072 | 0.00681632 | 0.0007 | 0.114 |
| ENSMFAG00000033450 | PLEKHM3 | -0.429858262 | 0.016662258 | 0.0009 | 0.119 |
| ENSMFAG00000044936 | SBF2 | -0.158640649 | 0.002270581 | 0.0009 | 0.119 |
| ENSMFAG00000040866 | ZNF701 | -0.324856057 | 0.009568627 | 0.0009 | 0.121 |
| ENSMFAG00000000676 | HLA-DRB5 | -0.5576798 | 0.028363565 | 0.0009 | 0.124 |
| ENSMFAG00000003553 | BRSK1 | 0.60144957 | 0.033276339 | 0.0010 | 0.129 |
| ENSMFAG00000030934 | PTPN12 | -0.23425123 | 0.005127584 | 0.0011 | 0.129 |
| ENSMFAG00000063827 | PTAFR | -0.639867736 | 0.03832258 | 0.0011 | 0.129 |
| ENSMFAG00000003987 | FAM149B1 | -0.145252651 | 0.001987178 | 0.0011 | 0.129 |
| ENSMFAG00000026741 | SCARA3 | 0.382880041 | 0.013711854 | 0.0011 | 0.129 |
| ENSMFAG00000044300 | NDRG1 | -0.229890574 | 0.004948103 | 0.0011 | 0.129 |
| ENSMFAG00000001892 | DQX1 | 0.152302077 | 0.002166778 | 0.0011 | 0.129 |
| ENSMFAG00000007199 | MIR193A | 1.510456572 | 0.211284684 | 0.0010 | 0.129 |
| ENSMFAG00000034193 | SLC66A3 | -0.221361492 | 0.004603918 | 0.0011 | 0.129 |
| ENSMFAG00000038160 | PTGES | -0.610469304 | 0.035195093 | 0.0011 | 0.129 |
| ENSMFAG00000038671 | LSR | -0.430868055 | 0.01747587 | 0.0011 | 0.129 |
| ENSMFAG00000042155 | TTC27 | -0.235585821 | 0.005191576 | 0.0011 | 0.129 |
| ENSMFAG00000045477 | NA | 0.872787338 | 0.071886967 | 0.0011 | 0.129 |
| ENSMFAG00000057027 | SNORD62 | 1.084003773 | 0.109918876 | 0.0011 | 0.129 |
| ENSMFAG00000006879 | SNORA5B | 0.829237854 | 0.066354379 | 0.0013 | 0.140 |
| ENSMFAG00000043819 | POMT2 | 0.206128763 | 0.004093959 | 0.0013 | 0.140 |
| ENSMFAG00000065768 | NA | -0.580717257 | 0.032381785 | 0.0013 | 0.140 |
| ENSMFAG00000043878 | SLX4 | 0.221004827 | 0.004703886 | 0.0013 | 0.140 |
| ENSMFAG00000042110 | ASB2 | 0.261263175 | 0.00667677 | 0.0014 | 0.141 |
| ENSMFAG00000044114 | ZCWPW1 | -0.308768629 | 0.009244886 | 0.0013 | 0.141 |
| ENSMFAG00000000932 | FNDC1 | 1.070938414 | 0.111414119 | 0.0013 | 0.141 |
| ENSMFAG00000030158 | FKBP5 | -0.763202035 | 0.057152188 | 0.0014 | 0.141 |
| ENSMFAG00000041231 | GALNT18 | 0.278547348 | 0.007587215 | 0.0014 | 0.141 |
| ENSMFAG00000048779 | SRSF12 | 0.500337549 | 0.024506866 | 0.0014 | 0.141 |
| ENSMFAG00000054921 | NA | -0.515908715 | 0.025986136 | 0.0014 | 0.141 |
| ENSMFAG00000039564 | ADGRE1 | -0.843066152 | 0.069642131 | 0.0014 | 0.141 |
| ENSMFAG00000039588 | GAREM1 | 0.245367698 | 0.005881318 | 0.0014 | 0.141 |
| ENSMFAG00000002430 | NA | -0.700925208 | 0.048340999 | 0.0014 | 0.142 |
| ENSMFAG00000035657 | NA | -0.194555817 | 0.003750909 | 0.0015 | 0.145 |
| ENSMFAG00000035726 | MAGIX | 0.28448925 | 0.008026437 | 0.0015 | 0.145 |
| ENSMFAG00000035422 | METTL1 | 0.229681712 | 0.005270788 | 0.0016 | 0.150 |
| ENSMFAG00000002740 | SP4 | -0.254374299 | 0.006501777 | 0.0016 | 0.152 |
| ENSMFAG00000031792 | TFPT | 0.20865648 | 0.004367791 | 0.0016 | 0.152 |
| ENSMFAG00000031423 | BMX | -0.642428113 | 0.041715164 | 0.0017 | 0.154 |
| ENSMFAG00000036236 | AMIGO2 | 0.341911006 | 0.011824609 | 0.0017 | 0.154 |
| ENSMFAG00000036309 | NA | 0.150208154 | 0.002303169 | 0.0017 | 0.155 |
| ENSMFAG00000044465 | GBP7 | -0.518239094 | 0.027360301 | 0.0017 | 0.155 |
| ENSMFAG00000065284 | NA | -0.300805908 | 0.009251191 | 0.0018 | 0.155 |
| ENSMFAG00000020572 | PLCE1 | -0.330842199 | 0.011127587 | 0.0017 | 0.155 |
| ENSMFAG00000038135 | NA | -0.171173343 | 0.002998702 | 0.0018 | 0.155 |
| ENSMFAG00000000325 | NA | 0.26545212 | 0.007171729 | 0.0017 | 0.155 |
| ENSMFAG00000037510 | MILR1 | -0.614851176 | 0.038546055 | 0.0017 | 0.155 |
| ENSMFAG00000043886 | NA | -0.17088182 | 0.003003214 | 0.0018 | 0.158 |
| ENSMFAG00000032370 | USPL1 | -0.164634148 | 0.002793617 | 0.0018 | 0.158 |
| ENSMFAG00000039386 | KLHDC3 | 0.115141267 | 0.001371246 | 0.0019 | 0.160 |
| ENSMFAG00000040953 | CAMK1 | 0.162262489 | 0.002730101 | 0.0019 | 0.161 |
| ENSMFAG00000048362 | VAMP3 | -0.1843418 | 0.003539526 | 0.0019 | 0.162 |
| ENSMFAG00000043340 | NA | 0.30852879 | 0.009901885 | 0.0019 | 0.162 |
| ENSMFAG00000001813 | TNN | -0.429991408 | 0.019412356 | 0.0020 | 0.165 |
| ENSMFAG00000003261 | C7 | -0.711584651 | 0.053430942 | 0.0021 | 0.165 |
| ENSMFAG00000031322 | FAXDC2 | -0.183627989 | 0.003539004 | 0.0020 | 0.165 |
| ENSMFAG00000041326 | CCDC181 | -0.221406263 | 0.005195456 | 0.0021 | 0.165 |
| ENSMFAG00000001306 | F13A1 | -0.883886079 | 0.082791618 | 0.0021 | 0.165 |
| ENSMFAG00000003083 | GPN3 | -0.192753123 | 0.00391945 | 0.0021 | 0.165 |
| ENSMFAG00000061462 | DEPP1 | 0.418232176 | 0.018357748 | 0.0020 | 0.165 |
| ENSMFAG00000035994 | SLC44A2 | -0.207266173 | 0.004545179 | 0.0021 | 0.165 |
| ENSMFAG00000041207 | UBB | 0.147695671 | 0.002296283 | 0.0021 | 0.165 |
| ENSMFAG00000063758 | NA | 0.493602128 | 0.025758956 | 0.0021 | 0.165 |
| ENSMFAG00000053142 | NA | 0.711231327 | 0.053745559 | 0.0022 | 0.165 |
| ENSMFAG00000002597 | IPO5 | -0.197292824 | 0.004192159 | 0.0023 | 0.176 |
| ENSMFAG00000003675 | SNX32 | 0.285823706 | 0.008859443 | 0.0024 | 0.177 |
| ENSMFAG00000042438 | NSMCE2 | -0.240449199 | 0.006257112 | 0.0024 | 0.177 |
| ENSMFAG00000057218 | NA | -1.153892542 | 0.143919134 | 0.0024 | 0.177 |
| ENSMFAG00000032728 | PSPC1 | 0.210473224 | 0.00480749 | 0.0024 | 0.177 |
| ENSMFAG00000039660 | C2orf15 | -0.255644303 | 0.007076314 | 0.0024 | 0.177 |
| ENSMFAG00000043983 | NA | 0.119159197 | 0.001565085 | 0.0026 | 0.184 |
| ENSMFAG00000001155 | NCKAP1L | -0.404308725 | 0.018100153 | 0.0027 | 0.184 |
| ENSMFAG00000003827 | RBM14 | 0.141937162 | 0.00221797 | 0.0026 | 0.184 |
| ENSMFAG00000021023 | SNRPA1 | -0.140825259 | 0.002196934 | 0.0027 | 0.184 |
| ENSMFAG00000045399 | NXF1 | 0.17252101 | 0.003274918 | 0.0026 | 0.184 |
| ENSMFAG00000048625 | NA | 0.579612294 | 0.037123844 | 0.0026 | 0.184 |
| ENSMFAG00000048993 | CLDN23 | 0.446613987 | 0.021978466 | 0.0026 | 0.184 |
| ENSMFAG00000060890 | NA | -0.328089664 | 0.011833361 | 0.0026 | 0.184 |
| ENSMFAG00000046112 | PLK1 | 0.318795763 | 0.011219487 | 0.0026 | 0.184 |
| ENSMFAG00000003879 | KLHDC1 | -0.244158321 | 0.006626076 | 0.0027 | 0.185 |
| ENSMFAG00000002882 | FAM32A | -0.157727246 | 0.002765232 | 0.0027 | 0.185 |
| ENSMFAG00000040523 | TMEM260 | -0.22929219 | 0.005863589 | 0.0027 | 0.186 |
| ENSMFAG00000054323 | NA | -0.237367282 | 0.006292559 | 0.0028 | 0.186 |
| ENSMFAG00000002617 | KLHL29 | 0.275941084 | 0.008507352 | 0.0028 | 0.186 |
| ENSMFAG00000058163 | NA | 0.496466542 | 0.027608985 | 0.0028 | 0.187 |
| ENSMFAG00000002826 | DPH6 | -0.351886814 | 0.013976639 | 0.0029 | 0.187 |
| ENSMFAG00000004660 | ASH1L | -0.205413414 | 0.004761245 | 0.0029 | 0.187 |
| ENSMFAG00000007433 | NA | 0.182026853 | 0.003729527 | 0.0029 | 0.187 |
| ENSMFAG00000035734 | NPC2 | -0.289983887 | 0.00948681 | 0.0029 | 0.187 |
| ENSMFAG00000056146 | NA | 0.743402393 | 0.062006043 | 0.0028 | 0.187 |
| ENSMFAG00000043421 | SREBF2 | -0.239333088 | 0.00645411 | 0.0029 | 0.187 |
| ENSMFAG00000002605 | NA | -0.2991988 | 0.010191436 | 0.0030 | 0.192 |
| ENSMFAG00000007057 | RAC3 | 0.624465326 | 0.04442608 | 0.0030 | 0.192 |
| ENSMFAG00000030901 | DCXR | 0.226416432 | 0.005827917 | 0.0030 | 0.192 |
| ENSMFAG00000029647 | SNX8 | -0.18828568 | 0.004048348 | 0.0031 | 0.193 |
| ENSMFAG00000001652 | SNAP25 | 0.522245762 | 0.031174393 | 0.0031 | 0.193 |
| ENSMFAG00000035423 | MAP1LC3A | 0.241826744 | 0.006706286 | 0.0031 | 0.195 |
| ENSMFAG00000003982 | PLPP5 | 0.189455121 | 0.004143697 | 0.0032 | 0.199 |
| ENSMFAG00000051510 | BHLHE41 | -0.458977492 | 0.024322777 | 0.0033 | 0.199 |

**Supplementary Table 3. Canonical pathways identified by Ingenuity Pathway Analysis from 174 differentially expressed genes with FDRs < 0.2 in iliac arteries.** Ratio indicates the number of target genes in the dataset divided by the total number of genes in the pathway.

| **Canonical Pathways** | **-log(p-value)** | | **Ratio** | **Molecules** |
| --- | --- | --- | --- | --- |
| Eukaryotic Translation Initiation | | 4.21 | 0.0492 | EIF3H,EIF4B,PABPC1,RPL15,RPL6,RPS4X |
| mTOR Signaling | | 3.71 | 0.0327 | AKT1S1,DIRAS3,EIF3H,EIF4B,PIK3R1,RAC3,RPS4X |
| Signaling by PDGF | | 3.52 | 0.069 | COL5A2,PDGFB,PIK3R1,PTPN12 |
| Sphingosine-1-phosphate Signaling | | 3.22 | 0.041 | DIRAS3,PDGFB,PIK3R1,PLCE1,RAC3 |
| Mitophagy | | 3.22 | 0.1 | MAP1LC3A,TOMM20,UBB |
| Plasma lipoprotein assembly, remodeling, and clearance | | 3.07 | 0.0526 | LDLRAP1,LSR,NPC2,UBB |
| EIF2 Signaling | | 2.73 | 0.026 | EIF3H,PABPC1,PIK3R1,RPL15,RPL6,RPS4X |
| Regulation of mRNA stability by proteins that bind AU-rich elements | | 2.73 | 0.0682 | ANP32A,PABPC1,UBB |
| Signaling by ERBB2 | | 2.57 | 0.06 | PIK3R1,PTPN12,UBB |
| Signaling by EGFR | | 2.57 | 0.06 | PIK3R1,PTPN12,UBB |
| Glioblastoma Multiforme Signaling | | 2.55 | 0.0289 | DIRAS3,PDGFB,PIK3R1,PLCE1,RAC3 |
| Signaling by NTRK3 (TRKC) | | 2.41 | 0.118 | NTRK3,PIK3R1 |
| RAN Signaling | | 2.41 | 0.118 | IPO5,RANGAP1 |
| Dermatan Sulfate Degradation (Metazoa) | | 2.41 | 0.118 | HYAL3,IDS |
| Nonsense-Mediated Decay (NMD) | | 2.38 | 0.0342 | PABPC1,RPL15,RPL6,RPS4X |
| Collagen biosynthesis and modifying enzymes | | 2.21 | 0.0448 | COL15A1,COL5A2,PLOD3 |
| Neutrophil Extracellular Trap Signaling Pathway | | 2.15 | 0.0175 | COL15A1,COL5A2,PIK3R1,PLCE1,RAC3,RIPK3,TOMM20 |
| Glioma Invasiveness Signaling | | 2.08 | 0.04 | DIRAS3,PIK3R1,RAC3 |
| Cholesterol biosynthesis | | 2.08 | 0.08 | ACAT2,SREBF2 |
| Thrombin Signaling | | 2.06 | 0.022 | CAMK1,DIRAS3,PIK3R1,PLCE1,RAC3 |
| Signaling by MET | | 2.01 | 0.038 | COL5A2,PIK3R1,UBB |
| PTEN Signaling | | 2 | 0.0265 | MAGIX,NTRK3,PIK3R1,RAC3 |
| Renal Cell Carcinoma Signaling | | 1.98 | 0.037 | PDGFB,PIK3R1,UBB |
| MTOR signalling | | 1.98 | 0.0714 | AKT1S1,EIF4B |
| PI Metabolism | | 1.97 | 0.0366 | BMX,PIK3R1,SBF2 |
| Microautophagy Signaling Pathway | | 1.91 | 0.025 | COL15A1,COL5A2,MAP1LC3A,TOMM20 |
| Pancreatic Secretion Signaling Pathway | | 1.9 | 0.0201 | AKT1S1,PIK3R1,PLCE1,SNAP25,VAMP3 |
| Signaling by CSF1 (M-CSF) in myeloid cells | | 1.89 | 0.0645 | PIK3R1,UBB |
| Toll Like Receptor 3 (TLR3) Cascade | | 1.87 | 0.0625 | RIPK3,UBB |
| RIPK1-mediated regulated necrosis | | 1.87 | 0.0625 | RIPK3,UBB |
| Eukaryotic Translation Termination | | 1.81 | 0.0319 | RPL15,RPL6,RPS4X |
| Fcγ Receptor-mediated Phagocytosis in Macrophages and Monocytes | | 1.81 | 0.0319 | PIK3R1,RAC3,VAMP3 |
| Eukaryotic Translation Elongation | | 1.8 | 0.0316 | RPL15,RPL6,RPS4X |
| Netrin Signaling | | 1.78 | 0.0229 | PIK3R1,RAC3,SNAP25,VAMP3 |
| Proline catabolism | | 1.78 | 0.333 | PRODH |
| SUMOylation of nuclear envelope proteins | | 1.78 | 0.333 | RANGAP1 |
| Diphthamide Biosynthesis | | 1.78 | 0.333 | DPH6 |
| Proline Degradation | | 1.78 | 0.333 | PRODH |
| FLT3 Signaling | | 1.77 | 0.0556 | PIK3R1,UBB |
| MyD88-independent TLR4 cascade | | 1.75 | 0.0541 | RIPK3,UBB |
| Fanconi Anemia Pathway | | 1.73 | 0.0526 | SLX4,UBB |
| Insulin Secretion Signaling Pathway | | 1.72 | 0.0181 | PABPC1,PIK3R1,PLCE1,SNAP25,VAMP3 |
| Response of EIF2AK4 (GCN2) to amino acid deficiency | | 1.71 | 0.0291 | RPL15,RPL6,RPS4X |
| Neuropathic Pain Signaling in Dorsal Horn Neurons | | 1.71 | 0.0291 | CAMK1,PIK3R1,PLCE1 |
| Sumoylation Pathway | | 1.71 | 0.0291 | DIRAS3,RAC3,RANGAP1 |
| Chronic Myeloid Leukemia Signaling | | 1.7 | 0.0179 | AKT1S1,PIK3R1,PLCE1,PLK1,RAC3 |
| Regulation of eIF4 and p70S6K Signaling | | 1.7 | 0.0216 | EIF3H,PABPC1,PIK3R1,RPS4X |
| Signaling by FGFR3 | | 1.68 | 0.05 | PIK3R1,UBB |
| Extracellular matrix organization | | 1.67 | 0.0283 | COL5A2,PDGFB,TNN |
| Signaling by FGFR4 | | 1.66 | 0.0488 | PIK3R1,UBB |
| Selenoamino acid metabolism | | 1.66 | 0.028 | RPL15,RPL6,RPS4X |
| Cargo recognition for clathrin-mediated endocytosis | | 1.66 | 0.028 | LDLRAP1,UBB,VAMP3 |
| Phenylethylamine Degradation I | | 1.66 | 0.25 | ALDH3A2 |
| Collagen chain trimerization | | 1.61 | 0.0455 | COL15A1,COL5A2 |
| SRP-dependent cotranslational protein targeting to membrane | | 1.58 | 0.0261 | RPL15,RPL6,RPS4X |
| Cytosolic sensors of pathogen-associated DNA | | 1.57 | 0.0435 | RIPK3,UBB |
| Role of Osteoclasts in Rheumatoid Arthritis Signaling Pathway | | 1.53 | 0.0161 | COL15A1,COL5A2,DIRAS3,PIK3R1,RAC3 |
| Clathrin-mediated Endocytosis Signaling | | 1.53 | 0.0191 | LDLRAP1,PDGFB,PIK3R1,UBB |
| HIF1α Signaling | | 1.52 | 0.019 | CAMK1,PDGFB,PIK3R1,RAC3 |
| Integrin Signaling | | 1.5 | 0.0187 | DIRAS3,PDGFB,PIK3R1,RAC3 |
| Signaling by FGFR1 | | 1.5 | 0.04 | PIK3R1,UBB |
| Autophagy | | 1.48 | 0.0184 | AKT1S1,MAP1LC3A,PDGFB,PIK3R1 |
| Glioma Signaling | | 1.47 | 0.0236 | CAMK1,PDGFB,PIK3R1 |
| Apoptotic execution phase | | 1.47 | 0.0385 | BMX,HMGB2 |
| Glutaminergic Receptor Signaling Pathway (Enhanced) | | 1.45 | 0.0153 | AKT1S1,PIK3R1,PLCE1,SNAP25,VAMP3 |
| 14-3-3-mediated Signaling | | 1.45 | 0.0233 | AKT1S1,PIK3R1,PLCE1 |
| GP6 Signaling Pathway | | 1.45 | 0.0233 | COL15A1,COL5A2,PIK3R1 |
| Endocannabinoid Developing Neuron Pathway | | 1.45 | 0.0233 | AKT1S1,PIK3R1,RAC3 |
| Clathrin-mediated endocytosis | | 1.45 | 0.0231 | LDLRAP1,UBB,VAMP3 |
| Myelination Signaling Pathway | | 1.44 | 0.0152 | AKT1S1,NDRG1,PDGFB,PIK3R1,SREBF2 |
| Deadenylation-dependent mRNA decay | | 1.43 | 0.0364 | EIF4B,PABPC1 |
| Interleukin-3, Interleukin-5 and GM-CSF signaling | | 1.43 | 0.0364 | PIK3R1,UBB |
| RHO GTPase cycle | | 1.4 | 0.0133 | ALDH3A2,AMIGO2,NCKAP1L,PIK3R1,RAC3,VAMP3 |
| Superpathway of Inositol Phosphate Compounds | | 1.37 | 0.0169 | PALD1,PIK3R1,PLCE1,PTPN12 |
| DHCR24 Signaling Pathway | | 1.37 | 0.0216 | PDGFB,PIK3R1,SREBF2 |
| Signaling by ERBB4 | | 1.37 | 0.0339 | PIK3R1,UBB |
| PIP3 activates AKT signaling | | 1.37 | 0.0214 | AKT1S1,PDGFB,PIK3R1 |
| NRF2-mediated Oxidative Stress Response | | 1.35 | 0.0167 | ERP29,FKBP5,PIK3R1,UBB |
| Collagen degradation | | 1.35 | 0.0328 | COL15A1,COL5A2 |
| Assembly of collagen fibrils and other multimeric structures | | 1.35 | 0.0328 | COL15A1,COL5A2 |
| Semaphorin Signaling in Neurons | | 1.35 | 0.0328 | DIRAS3,RAC3 |
| Hereditary Breast Cancer Signaling | | 1.34 | 0.0208 | MSH2,PIK3R1,UBB |
| Actin Cytoskeleton Signaling | | 1.32 | 0.0163 | NCKAP1L,PDGFB,PIK3R1,RAC3 |
| Mitochondrial protein import | | 1.31 | 0.0312 | CHCHD7,TOMM20 |

**Supplementary Table 4. Differentially expressed genes as a function of social status (continuous relative rank score) in carotid arteries at an FDR threshold of 0.2.** Beta and "SE" refer to the beta coefficient and standard error, respectively. Bold = FDR < 0.05. Positive beta means upregulation and negative beta means downregulation in the subordinate cohort relative to the dominant cohort. Transcripts are organized from smallest to largest FDR. Bold indicates FDR values <0.05.

| **Gene ID** | **Gene Names** | **beta** | **SE** | **p** | **FDR** |
| --- | --- | --- | --- | --- | --- |
| ENSMFAG00000059809 | **LncRNA** | 0.968 | 0.029 | 1.56E-08 | **0.0002** |
| ENSMFAG00000034264 | **KCNQ4** | 0.462 | 0.010 | 2.28E-06 | **0.0132** |
| ENSMFAG00000033220 | **IRAK1BP1** | -0.457 | 0.010 | 3.79E-06 | **0.0146** |
| ENSMFAG00000040584 | **TNKS1BP1** | 0.372 | 0.007 | 5.83E-06 | **0.0164** |
| ENSMFAG00000024723 | **KIAA0513** | -0.659 | 0.022 | 7.08E-06 | **0.0164** |
| ENSMFAG00000050714 | **LncRNA** | -0.518 | 0.014 | 8.71E-06 | **0.0168** |
| ENSMFAG00000042264 | **KCNIP3** | 0.392 | 0.008 | 1.73E-05 | **0.0285** |
| ENSMFAG00000041414 | **CSNK1D** | 0.150 | 0.001 | 2.29E-05 | **0.0331** |
| ENSMFAG00000051035 | **SMIM15** | -0.255 | 0.004 | 3.34E-05 | **0.0379** |
| ENSMFAG00000034327 | **PNPLA7** | 0.410 | 0.010 | 3.56E-05 | **0.0379** |
| ENSMFAG00000037937 | **INPPL1** | 0.245 | 0.004 | 3.61E-05 | **0.0379** |
| ENSMFAG00000002257 | **PSMD14** | -0.239 | 0.003 | 4.21E-05 | **0.0403** |
| ENSMFAG00000000541 | **TOPORS** | -0.150 | 0.001 | 4.53E-05 | **0.0403** |
| ENSMFAG00000030106 | **ARPC2** | -0.243 | 0.004 | 5.06E-05 | **0.0412** |
| ENSMFAG00000041314 | **RAD9A** | 0.342 | 0.007 | 5.37E-05 | **0.0412** |
| ENSMFAG00000053865 | **A0A7N9CS45** | 0.746 | 0.035 | 5.91E-05 | **0.0412** |
| ENSMFAG00000043700 | **F10** | 0.683 | 0.029 | 6.06E-05 | **0.0412** |
| ENSMFAG00000033963 | **STIM1** | 0.196 | 0.002 | 7.33E-05 | **0.0470** |
| ENSMFAG00000001982 | ATN1 | 0.167 | 0.002 | 8.86E-05 | 0.0504 |
| ENSMFAG00000002767 | ATG2A | 0.222 | 0.003 | 9.41E-05 | 0.0504 |
| ENSMFAG00000034049 | URGCP | 0.165 | 0.002 | 9.83E-05 | 0.0504 |
| ENSMFAG00000021023 | SNRPA1 | -0.210 | 0.003 | 9.87E-05 | 0.0504 |
| ENSMFAG00000003364 | AP3S1 | -0.226 | 0.003 | 0.0001 | 0.0504 |
| ENSMFAG00000025261 | CDC42EP2 | 0.638 | 0.027 | 0.0001 | 0.0517 |
| ENSMFAG00000064434 | NA | 1.553 | 0.162 | 0.0001 | 0.0517 |
| ENSMFAG00000035698 | NA | 0.635 | 0.027 | 0.0001 | 0.0541 |
| ENSMFAG00000045158 | METTL13 | -0.335 | 0.008 | 0.0001 | 0.0592 |
| ENSMFAG00000031449 | MANSC1 | -0.260 | 0.005 | 0.0002 | 0.0606 |
| ENSMFAG00000002383 | OLFML2A | 0.745 | 0.039 | 0.0002 | 0.0606 |
| ENSMFAG00000033122 | SF1 | 0.165 | 0.002 | 0.0002 | 0.0606 |
| ENSMFAG00000043717 | GLUD1 | -0.347 | 0.008 | 0.0002 | 0.0606 |
| ENSMFAG00000055884 | NA | 0.380 | 0.010 | 0.0002 | 0.0656 |
| ENSMFAG00000045229 | MST1R | 0.299 | 0.006 | 0.0002 | 0.0675 |
| ENSMFAG00000033745 | SRRT | 0.202 | 0.003 | 0.0002 | 0.0675 |
| ENSMFAG00000031813 | NCKAP5L | 0.221 | 0.004 | 0.0002 | 0.0779 |
| ENSMFAG00000002488 | RAI1 | 0.296 | 0.007 | 0.0002 | 0.0779 |
| ENSMFAG00000035471 | SDR39U1 | 0.212 | 0.003 | 0.0003 | 0.0779 |
| ENSMFAG00000033956 | DENND6B | 0.298 | 0.007 | 0.0003 | 0.0779 |
| ENSMFAG00000053669 | NA | -0.211 | 0.003 | 0.0003 | 0.0779 |
| ENSMFAG00000048292 | NA | 1.136 | 0.097 | 0.0003 | 0.0779 |
| ENSMFAG00000035432 | ZNF335 | 0.302 | 0.007 | 0.0003 | 0.0779 |
| ENSMFAG00000000131 | SLC2A8 | 0.526 | 0.021 | 0.0003 | 0.0853 |
| ENSMFAG00000031838 | SYAP1 | -0.272 | 0.006 | 0.0003 | 0.0853 |
| ENSMFAG00000038006 | ASAP3 | 0.381 | 0.011 | 0.0003 | 0.0853 |
| ENSMFAG00000035524 | BFSP1 | 0.954 | 0.071 | 0.0004 | 0.0880 |
| ENSMFAG00000062474 | NA | 0.284 | 0.006 | 0.0004 | 0.0880 |
| ENSMFAG00000050274 | NA | -0.731 | 0.042 | 0.0004 | 0.0880 |
| ENSMFAG00000056275 | Metazoa_SRP | 0.447 | 0.016 | 0.0004 | 0.0880 |
| ENSMFAG00000039706 | SAFB2 | 0.229 | 0.004 | 0.0004 | 0.0880 |
| ENSMFAG00000002016 | NA | 0.442 | 0.015 | 0.0004 | 0.0880 |
| ENSMFAG00000042359 | MDM2 | -0.359 | 0.010 | 0.0004 | 0.0880 |
| ENSMFAG00000056949 | RWDD2A | -0.309 | 0.008 | 0.0004 | 0.0880 |
| ENSMFAG00000030440 | TAOK2 | 0.172 | 0.002 | 0.0004 | 0.0880 |
| ENSMFAG00000003740 | DHX29 | -0.176 | 0.002 | 0.0004 | 0.0906 |
| ENSMFAG00000040263 | ANKRD11 | 0.131 | 0.001 | 0.0004 | 0.0919 |
| ENSMFAG00000000751 | PLGRKT | -0.273 | 0.006 | 0.0005 | 0.0956 |
| ENSMFAG00000031946 | BIN1 | 0.338 | 0.009 | 0.0005 | 0.0956 |
| ENSMFAG00000064072 | Metazoa_SRP | 0.934 | 0.072 | 0.0005 | 0.0956 |
| ENSMFAG00000040724 | GABBR1 | 0.309 | 0.008 | 0.0005 | 0.0956 |
| ENSMFAG00000003394 | SMG6 | 0.174 | 0.002 | 0.0005 | 0.0956 |
| ENSMFAG00000032166 | TSC2 | 0.228 | 0.004 | 0.0005 | 0.0956 |
| ENSMFAG00000035726 | MAGIX | 0.417 | 0.014 | 0.0005 | 0.0956 |
| ENSMFAG00000001007 | PANK4 | 0.285 | 0.007 | 0.0005 | 0.0956 |
| ENSMFAG00000001381 | BRF1 | 0.295 | 0.007 | 0.0005 | 0.0956 |
| ENSMFAG00000002256 | C1GALT1C1 | -0.241 | 0.005 | 0.0005 | 0.0956 |
| ENSMFAG00000001706 | PXMP2 | 0.562 | 0.026 | 0.0005 | 0.0961 |
| ENSMFAG00000049216 | SLC25A51 | -0.156 | 0.002 | 0.0006 | 0.0961 |
| ENSMFAG00000033857 | TMEM50A | -0.153 | 0.002 | 0.0006 | 0.0984 |
| ENSMFAG00000010699 | NA | -0.934 | 0.074 | 0.0006 | 0.1005 |
| ENSMFAG00000049336 | NA | 0.581 | 0.029 | 0.0006 | 0.1045 |
| ENSMFAG00000001553 | YIPF5 | -0.156 | 0.002 | 0.0007 | 0.1062 |
| ENSMFAG00000043623 | NCOA5 | 0.150 | 0.002 | 0.0007 | 0.1074 |
| ENSMFAG00000045446 | CNTN3 | -0.710 | 0.044 | 0.0007 | 0.1074 |
| ENSMFAG00000002561 | ARHGAP18 | -0.644 | 0.036 | 0.0007 | 0.1136 |
| ENSMFAG00000038333 | TMEM259 | 0.212 | 0.004 | 0.0007 | 0.1137 |
| ENSMFAG00000000298 | NA | -0.668 | 0.039 | 0.0008 | 0.1148 |
| ENSMFAG00000001738 | NA | 1.005 | 0.090 | 0.0008 | 0.1148 |
| ENSMFAG00000047226 | NA | -0.534 | 0.025 | 0.0008 | 0.1148 |
| ENSMFAG00000042055 | TRIM13 | -0.306 | 0.008 | 0.0008 | 0.1148 |
| ENSMFAG00000032121 | CLASRP | 0.249 | 0.006 | 0.0008 | 0.1148 |
| ENSMFAG00000042056 | NA | -0.201 | 0.004 | 0.0008 | 0.1148 |
| ENSMFAG00000053132 | NA | 0.223 | 0.004 | 0.0009 | 0.1199 |
| ENSMFAG00000037527 | SLC8A2 | 0.735 | 0.049 | 0.0009 | 0.1199 |
| ENSMFAG00000032985 | ATP6V1H | -0.174 | 0.003 | 0.0009 | 0.1228 |
| ENSMFAG00000026128 | YTHDF2 | -0.151 | 0.002 | 0.0009 | 0.1228 |
| ENSMFAG00000003781 | CBFA2T3 | 0.592 | 0.032 | 0.0009 | 0.1228 |
| ENSMFAG00000041098 | ARL2 | 0.187 | 0.003 | 0.0010 | 0.1298 |
| ENSMFAG00000031150 | BAHD1 | 0.252 | 0.006 | 0.0010 | 0.1298 |
| ENSMFAG00000051177 | NA | 0.619 | 0.036 | 0.0010 | 0.1345 |
| ENSMFAG00000045827 | NA | 1.228 | 0.141 | 0.0011 | 0.1379 |
| ENSMFAG00000043642 | DBR1 | -0.256 | 0.006 | 0.0011 | 0.1388 |
| ENSMFAG00000031035 | ARAF | 0.142 | 0.002 | 0.0011 | 0.1388 |
| ENSMFAG00000002085 | MBD6 | 0.206 | 0.004 | 0.0011 | 0.1388 |
| ENSMFAG00000002539 | GTF2E1 | -0.269 | 0.007 | 0.0011 | 0.1388 |
| ENSMFAG00000044437 | ACIN1 | 0.128 | 0.002 | 0.0012 | 0.1402 |
| ENSMFAG00000001206 | VPS13A | -0.410 | 0.016 | 0.0012 | 0.1402 |
| ENSMFAG00000032465 | SMAP1 | -0.266 | 0.007 | 0.0012 | 0.1402 |
| ENSMFAG00000034499 | SLC37A4 | -0.303 | 0.009 | 0.0012 | 0.1402 |
| ENSMFAG00000034069 | MEIS3 | 0.494 | 0.023 | 0.0012 | 0.1447 |
| ENSMFAG00000007411 | KCNS3 | -0.864 | 0.072 | 0.0013 | 0.1531 |
| ENSMFAG00000002737 | SH3BP5L | 0.204 | 0.004 | 0.0013 | 0.1531 |
| ENSMFAG00000001735 | SRI | -0.180 | 0.003 | 0.0014 | 0.1531 |
| ENSMFAG00000045997 | ARRDC4 | -0.664 | 0.043 | 0.0014 | 0.1531 |
| ENSMFAG00000032283 | AK8 | 0.664 | 0.043 | 0.0014 | 0.1531 |
| ENSMFAG00000035574 | NA | -0.507 | 0.025 | 0.0014 | 0.1531 |
| ENSMFAG00000053934 | NA | -0.263 | 0.007 | 0.0014 | 0.1531 |
| ENSMFAG00000002105 | LNPK | -0.225 | 0.005 | 0.0014 | 0.1531 |
| ENSMFAG00000041002 | PUS3 | -0.174 | 0.003 | 0.0014 | 0.1531 |
| ENSMFAG00000045568 | NA | 1.639 | 0.265 | 0.0014 | 0.1531 |
| ENSMFAG00000001271 | PACS1 | 0.145 | 0.002 | 0.0015 | 0.1531 |
| ENSMFAG00000046023 | FCER2 | 1.390 | 0.193 | 0.0015 | 0.1567 |
| ENSMFAG00000043892 | CD44 | -0.657 | 0.043 | 0.0015 | 0.1567 |
| ENSMFAG00000056563 | NA | 1.724 | 0.297 | 0.0016 | 0.1567 |
| ENSMFAG00000044799 | DGUOK | -0.268 | 0.007 | 0.0016 | 0.1567 |
| ENSMFAG00000055454 | NA | -0.654 | 0.043 | 0.0016 | 0.1567 |
| ENSMFAG00000045952 | RHOT2 | 0.209 | 0.004 | 0.0016 | 0.1583 |
| ENSMFAG00000031206 | MGRN1 | 0.204 | 0.004 | 0.0016 | 0.1584 |
| ENSMFAG00000029268 | NISCH | 0.251 | 0.006 | 0.0016 | 0.1584 |
| ENSMFAG00000035825 | NA | -0.203 | 0.004 | 0.0017 | 0.1606 |
| ENSMFAG00000046083 | TNK2 | 0.312 | 0.010 | 0.0017 | 0.1617 |
| ENSMFAG00000036346 | PRDX4 | -0.231 | 0.005 | 0.0017 | 0.1645 |
| ENSMFAG00000000861 | OAZ2 | 0.157 | 0.003 | 0.0018 | 0.1645 |
| ENSMFAG00000030098 | GPR146 | 0.707 | 0.051 | 0.0018 | 0.1645 |
| ENSMFAG00000037480 | NA | 0.317 | 0.010 | 0.0018 | 0.1645 |
| ENSMFAG00000042005 | SAP25 | 0.475 | 0.023 | 0.0018 | 0.1645 |
| ENSMFAG00000045340 | SLC38A7 | 0.265 | 0.007 | 0.0018 | 0.1645 |
| ENSMFAG00000040947 | PELI2 | -0.803 | 0.066 | 0.0018 | 0.1645 |
| ENSMFAG00000007545 | TMEFF1 | -0.363 | 0.014 | 0.0018 | 0.1645 |
| ENSMFAG00000036147 | ELOVL4 | -0.419 | 0.018 | 0.0018 | 0.1645 |
| ENSMFAG00000021292 | ZMAT3 | -0.530 | 0.029 | 0.0019 | 0.1645 |
| ENSMFAG00000003777 | PIK3C3 | -0.229 | 0.005 | 0.0019 | 0.1645 |
| ENSMFAG00000045078 | TEPSIN | 0.277 | 0.008 | 0.0019 | 0.1645 |
| ENSMFAG00000030989 | TJAP1 | 0.160 | 0.003 | 0.0019 | 0.1645 |
| ENSMFAG00000035383 | MRPS25 | 0.225 | 0.005 | 0.0019 | 0.1645 |
| ENSMFAG00000039690 | PWWP3A | 0.184 | 0.004 | 0.0019 | 0.1645 |
| ENSMFAG00000003526 | MRPL4 | 0.272 | 0.008 | 0.0020 | 0.1672 |
| ENSMFAG00000030713 | SLC39A9 | -0.161 | 0.003 | 0.0020 | 0.1672 |
| ENSMFAG00000042423 | ECHDC2 | 0.389 | 0.016 | 0.0020 | 0.1672 |
| ENSMFAG00000036760 | HPSE2 | -0.348 | 0.013 | 0.0020 | 0.1672 |
| ENSMFAG00000026117 | NID1 | 0.317 | 0.011 | 0.0021 | 0.1706 |
| ENSMFAG00000035388 | ATP11B | -0.275 | 0.008 | 0.0021 | 0.1707 |
| ENSMFAG00000034054 | TBCK | -0.331 | 0.012 | 0.0021 | 0.1707 |
| ENSMFAG00000047727 | ZFP3 | -0.359 | 0.014 | 0.0021 | 0.1707 |
| ENSMFAG00000045670 | ACBD3 | -0.158 | 0.003 | 0.0021 | 0.1707 |
| ENSMFAG00000055768 | NA | 0.286 | 0.009 | 0.0021 | 0.1707 |
| ENSMFAG00000036918 | CAAP1 | -0.262 | 0.007 | 0.0022 | 0.1708 |
| ENSMFAG00000038855 | SLC12A4 | 0.175 | 0.003 | 0.0022 | 0.1711 |
| ENSMFAG00000001451 | CDK7 | -0.257 | 0.007 | 0.0022 | 0.1731 |
| ENSMFAG00000036547 | NTNG1 | -0.696 | 0.052 | 0.0022 | 0.1731 |
| ENSMFAG00000033348 | NA | 0.614 | 0.041 | 0.0023 | 0.1731 |
| ENSMFAG00000043621 | TACR1 | 0.894 | 0.086 | 0.0023 | 0.1731 |
| ENSMFAG00000043828 | DENND2A | 0.709 | 0.054 | 0.0023 | 0.1731 |
| ENSMFAG00000038545 | ATP6AP2 | -0.200 | 0.004 | 0.0023 | 0.1731 |
| ENSMFAG00000001545 | CDC25B | 0.373 | 0.015 | 0.0023 | 0.1731 |
| ENSMFAG00000035901 | ARL6 | -0.506 | 0.028 | 0.0023 | 0.1731 |
| ENSMFAG00000045469 | XRCC3 | 0.237 | 0.006 | 0.0023 | 0.1731 |
| ENSMFAG00000033980 | ZNF449 | -0.348 | 0.013 | 0.0024 | 0.1732 |
| ENSMFAG00000043136 | NA | 0.191 | 0.004 | 0.0024 | 0.1732 |
| ENSMFAG00000002861 | DGKD | 0.324 | 0.011 | 0.0024 | 0.1746 |
| ENSMFAG00000035826 | ATP13A2 | 0.243 | 0.006 | 0.0024 | 0.1746 |
| ENSMFAG00000039202 | PNCK | 0.427 | 0.020 | 0.0024 | 0.1751 |
| ENSMFAG00000030830 | NA | 0.771 | 0.065 | 0.0025 | 0.1782 |
| ENSMFAG00000031325 | CCPG1 | -0.228 | 0.006 | 0.0025 | 0.1791 |
| ENSMFAG00000032377 | CCDC32 | -0.198 | 0.004 | 0.0026 | 0.1814 |
| ENSMFAG00000032785 | PAPSS1 | -0.232 | 0.006 | 0.0026 | 0.1814 |
| ENSMFAG00000002925 | NA | 0.211 | 0.005 | 0.0026 | 0.1833 |
| ENSMFAG00000027901 | DHRS7B | 0.192 | 0.004 | 0.0027 | 0.1836 |
| ENSMFAG00000033426 | ARMC10 | -0.250 | 0.007 | 0.0027 | 0.1836 |
| ENSMFAG00000000154 | SGSM2 | 0.292 | 0.009 | 0.0027 | 0.1836 |
| ENSMFAG00000000688 | NOTCH1 | 0.441 | 0.022 | 0.0028 | 0.1836 |
| ENSMFAG00000045591 | OSGEP | -0.229 | 0.006 | 0.0028 | 0.1836 |
| ENSMFAG00000002496 | PRSS53 | 0.290 | 0.009 | 0.0028 | 0.1836 |
| ENSMFAG00000043985 | COL5A3 | 0.709 | 0.056 | 0.0028 | 0.1836 |
| ENSMFAG00000039846 | EXOC8 | -0.252 | 0.007 | 0.0029 | 0.1836 |
| ENSMFAG00000036066 | GCLM | -0.373 | 0.016 | 0.0029 | 0.1836 |
| ENSMFAG00000004074 | TMEM45A | -0.490 | 0.027 | 0.0029 | 0.1836 |
| ENSMFAG00000000479 | ABCF3 | 0.164 | 0.003 | 0.0029 | 0.1836 |
| ENSMFAG00000022956 | ITGA7 | 0.353 | 0.014 | 0.0029 | 0.1836 |
| ENSMFAG00000039845 | NA | 0.325 | 0.012 | 0.0029 | 0.1836 |
| ENSMFAG00000002053 | RUNDC3B | -0.836 | 0.079 | 0.0029 | 0.1836 |
| ENSMFAG00000032235 | PDGFC | -0.291 | 0.010 | 0.0029 | 0.1836 |
| ENSMFAG00000032884 | SRCAP | 0.144 | 0.002 | 0.0029 | 0.1836 |
| ENSMFAG00000000240 | PHPT1 | 0.366 | 0.015 | 0.0029 | 0.1836 |
| ENSMFAG00000037495 | HMCN1 | -0.502 | 0.029 | 0.0030 | 0.1836 |
| ENSMFAG00000003266 | MGP | -0.569 | 0.037 | 0.0030 | 0.1836 |
| ENSMFAG00000027020 | UBE2O | 0.140 | 0.002 | 0.0030 | 0.1836 |
| ENSMFAG00000037245 | MED15 | 0.168 | 0.003 | 0.0030 | 0.1836 |
| ENSMFAG00000033566 | IL27 | 1.023 | 0.119 | 0.0030 | 0.1836 |
| ENSMFAG00000029240 | ADM5 | 0.693 | 0.055 | 0.0030 | 0.1836 |
| ENSMFAG00000033450 | PLEKHM3 | -0.561 | 0.036 | 0.0030 | 0.1836 |
| ENSMFAG00000036495 | USP40 | 0.202 | 0.005 | 0.0030 | 0.1836 |
| ENSMFAG00000039578 | AGTPBP1 | -0.303 | 0.010 | 0.0030 | 0.1836 |
| ENSMFAG00000033148 | DNAJC10 | -0.252 | 0.007 | 0.0031 | 0.1876 |
| ENSMFAG00000032206 | DLG4 | 0.277 | 0.009 | 0.0032 | 0.1884 |
| ENSMFAG00000039328 | MRE11 | -0.322 | 0.012 | 0.0032 | 0.1884 |
| ENSMFAG00000032191 | GRINA | 0.264 | 0.008 | 0.0032 | 0.1884 |
| ENSMFAG00000020124 | GLG1 | -0.118 | 0.002 | 0.0032 | 0.1884 |
| ENSMFAG00000058528 | NA | 1.622 | 0.304 | 0.0032 | 0.1884 |
| ENSMFAG00000030905 | NA | -0.142 | 0.002 | 0.0033 | 0.1884 |
| ENSMFAG00000040019 | ARHGEF10L | 0.242 | 0.007 | 0.0033 | 0.1884 |
| ENSMFAG00000019988 | TCEAL8 | -0.161 | 0.003 | 0.0033 | 0.1904 |
| ENSMFAG00000032537 | TBC1D16 | 0.257 | 0.008 | 0.0033 | 0.1905 |
| ENSMFAG00000001038 | NPY1R | 0.957 | 0.106 | 0.0033 | 0.1905 |
| ENSMFAG00000030593 | NA | -0.202 | 0.005 | 0.0034 | 0.1930 |
| ENSMFAG00000045367 | PBX2 | 0.143 | 0.002 | 0.0034 | 0.1930 |
| ENSMFAG00000046284 | PTP4A1 | -0.262 | 0.008 | 0.0035 | 0.1930 |
| ENSMFAG00000033260 | STAMBPL1 | -0.504 | 0.030 | 0.0035 | 0.1930 |
| ENSMFAG00000034360 | NACAD | 0.299 | 0.010 | 0.0035 | 0.1930 |
| ENSMFAG00000030551 | CDIP1 | 0.222 | 0.006 | 0.0035 | 0.1930 |
| ENSMFAG00000001398 | MIB2 | 0.223 | 0.006 | 0.0035 | 0.1930 |
| ENSMFAG00000029610 | TNFRSF10A | -0.510 | 0.030 | 0.0035 | 0.1930 |
| ENSMFAG00000043269 | LDLRAD3 | -0.445 | 0.023 | 0.0035 | 0.1931 |
| ENSMFAG00000040390 | LYRM7 | -0.405 | 0.019 | 0.0036 | 0.1931 |
| ENSMFAG00000039357 | TYMS | -0.510 | 0.031 | 0.0037 | 0.1974 |
| ENSMFAG00000029838 | TGS1 | -0.184 | 0.004 | 0.0037 | 0.1982 |
| ENSMFAG00000043096 | DKK3 | -0.732 | 0.064 | 0.0037 | 0.1996 |

**Supplementary Table 5. Canonical pathways identified by Ingenuity Pathway Analysis from 216 differentially expressed genes with FDRs < 0.2 in carotid arteries.** Ratio indicates the number of target genes in the dataset divided by the total number of genes in the pathway.

| **Canonical Pathways** | **-log(p-value)** | **Ratio** | **Molecules** |
| --- | --- | --- | --- |
| Reelin Signaling in Neurons | 2.39 | 0.0362 | ARHGEF10L,ARHGEF4,ARPC2,GRINA,PIK3C3 |
| Regulation of TP53 Activity through Phosphorylation | 2.26 | 0.043 | MDM2,MRE11,RAD9A,RPA3 |
| Mitochondrial translation | 2.21 | 0.0417 | MRPL1,MRPL4,MRPL47,MRPS25 |
| Salvage Pathways of Pyrimidine Ribonucleotides | 2.2 | 0.0412 | AK8,ARAF,CDK7,CSNK1D |
| Cell Cycle: G2/M DNA Damage Checkpoint Regulation | 2.17 | 0.0588 | CDC25B,CDK7,MDM2 |
| Glioblastoma Multiforme Signaling | 2.01 | 0.0292 | MDM2,PDGFC,PIK3C3,RHOT2,TSC2 |
| Circadian Clock | 1.99 | 0.0508 | CSNK1D,RAI1,TGS1 |
| HDR through Homologous Recombination (HRR) or Single Strand Annealing (SSA) | 1.98 | 0.0357 | MRE11,RAD9A,RPA3,XRCC3 |
| Pyrimidine Deoxyribonucleotides De Novo Biosynthesis I | 1.89 | 0.087 | AK8,TYMS |
| Pyridoxal 5'-phosphate Salvage Pathway | 1.88 | 0.0462 | ARAF,CDK7,CSNK1D |
| Sulfate Activation for Sulfonation | 1.82 | 0.5 | PAPSS1 |
| Pancreatic Adenocarcinoma Signaling | 1.81 | 0.0317 | MDM2,NOTCH1,PDGFC,PIK3C3 |
| Signaling by Rho Family GTPases | 1.79 | 0.0225 | ARHGEF4,ARPC2,CDC42EP2,ITGA7,PIK3C3,RHOT2 |
| TR/RXR Activation | 1.78 | 0.0312 | F10,MDM2,PDGFC,PIK3C3 |
| Cardiac conduction | 1.76 | 0.0308 | KCNIP3,SLC8A2,SRI,STIM1 |
| Clathrin-mediated endocytosis | 1.76 | 0.0308 | ARPC2,BIN1,SLC2A8,TACR1 |
| Glioma Invasiveness Signaling | 1.74 | 0.0411 | CD44,PIK3C3,RHOT2 |
| ID1 Signaling Pathway | 1.73 | 0.0249 | ARAF,MDM2,PDGFC,PIK3C3,TYMS |
| Hypoxia Signaling in the Cardiovascular System | 1.7 | 0.0395 | CSNK1D,MDM2,UBE2O |
| RAC Signaling | 1.69 | 0.0292 | ARPC2,CD44,ITGA7,PIK3C3 |
| Clathrin-mediated Endocytosis Signaling | 1.67 | 0.024 | AP3S1,ARPC2,MDM2,PDGFC,PIK3C3 |
| Role of p14/p19ARF in Tumor Suppression | 1.67 | 0.0667 | MDM2,PIK3C3 |
| HIF1α Signaling | 1.66 | 0.0239 | ARAF,MDM2,PDGFC,PIK3C3,SLC2A8 |
| MSP-RON Signaling in Cancer Cells Pathway | 1.66 | 0.0286 | ARAF,MST1R,PDGFC,PIK3C3 |
| Regulation of NFE2L2 gene expression | 1.65 | 0.333 | NOTCH1 |
| Glutathione Biosynthesis | 1.65 | 0.333 | GCLM |
| Integrin Signaling | 1.64 | 0.0236 | ARPC2,ITGA7,PIK3C3,RHOT2,TNK2 |
| Glycosaminoglycan metabolism | 1.64 | 0.0375 | CD44,HPSE2,PAPSS1 |
| Glutamate binding, activation of AMPA receptors and synaptic plasticity | 1.61 | 0.0625 | DLG4,MDM2 |
| PI Metabolism | 1.61 | 0.0366 | INPPL1,PIK3C3,PNPLA7 |
| RNA polymerase II transcribes snRNA genes | 1.6 | 0.0361 | CDK7,GTF2E1,SRRT |
| RHOGDI Signaling | 1.58 | 0.0227 | ARHGEF4,ARPC2,CD44,ITGA7,RHOT2 |
| Integrin cell surface interactions | 1.57 | 0.0353 | CD44,COL5A3,ITGA7 |
| Signaling by NOTCH2 | 1.56 | 0.0588 | FCER2,MIB2 |
| Platelet homeostasis | 1.56 | 0.0349 | SLC8A2,SRI,STIM1 |
| HIPPO signaling | 1.55 | 0.0345 | CD44,CSNK1D,DLG4 |
| PDGF Signaling | 1.55 | 0.0345 | INPPL1,PDGFC,PIK3C3 |
| Transcriptional Regulation by NPAS4 | 1.54 | 0.0571 | KCNIP3,MDM2 |
| Nucleotide Excision Repair Pathway | 1.54 | 0.0571 | CDK7,RPA3 |
| Molecular Mechanisms of Cancer | 1.52 | 0.014 | ARHGEF4,CDC25B,GABBR1,GPR146,ITGA7,MDM2,MRE11,NOTCH1,NPY1R,PIK3C3,RHOT2,TACR1 |
| Actin Nucleation by ARP-WASP Complex | 1.47 | 0.0323 | ARPC2,ITGA7,RHOT2 |
| HEY1 Signaling Pathway | 1.46 | 0.0248 | MDM2,NOTCH1,PDGFC,PIK3C3 |
| Transcriptional regulation by RUNX3 | 1.44 | 0.0312 | MDM2,NOTCH1,PSMD14 |
| Pyrimidine Ribonucleotides Interconversion | 1.43 | 0.05 | AK8,DHX29 |
| Signaling by MST1 | 1.43 | 0.2 | MST1R |
| dTMP De Novo Biosynthesis | 1.43 | 0.2 | TYMS |
| Actin Cytoskeleton Signaling | 1.41 | 0.0205 | ARHGEF4,ARPC2,ITGA7,PDGFC,PIK3C3 |
| p53 Signaling | 1.41 | 0.0306 | CSNK1D,MDM2,PIK3C3 |
| Intrinsic Prothrombin Activation Pathway | 1.39 | 0.0476 | COL5A3,F10 |
| S Phase | 1.39 | 0.03 | CDC25B,CDK7,PSMD14 |
| ATM Signaling | 1.39 | 0.03 | MDM2,MRE11,RAD9A |
| Neuropathic Pain Signaling in Dorsal Horn Neurons | 1.38 | 0.0297 | GRINA,PIK3C3,TACR1 |
| Pyrimidine Ribonucleotides De Novo Biosynthesis | 1.38 | 0.0465 | AK8,DHX29 |
| Hepatic Fibrosis Signaling Pathway | 1.37 | 0.0165 | ARAF,COL5A3,CSNK1D,ITGA7,PDGFC,PIK3C3,RHOT2 |
| Potassium Channels | 1.36 | 0.0291 | GABBR1,KCNQ4,KCNS3 |
| WNT/β-catenin Signaling | 1.36 | 0.023 | CD44,CSNK1D,DKK3,MDM2 |
| TBC/RABGAPs | 1.36 | 0.0455 | TBC1D16,TSC2 |
| Transport of inorganic cations/anions and amino acids/oligopeptides | 1.35 | 0.0288 | SLC12A4,SLC8A2,SRI |
| WNK Renal Signaling Pathway | 1.33 | 0.0283 | MDM2,PIK3C3,SLC12A4 |
| Extracellular matrix organization | 1.33 | 0.0283 | COL5A3,ITGA7,NID1 |
| DNA damage-induced 14-3-3σ Signaling | 1.32 | 0.0435 | MDM2,RAD9A |
| Tumor Microenvironment Pathway | 1.32 | 0.0223 | ARAF,CD44,PDGFC,PIK3C3 |
| Heme signaling | 1.31 | 0.0426 | RAI1,TGS1 |
| nNOS Signaling in Neurons | 1.31 | 0.0426 | DLG4,GRINA |

**Supplementary Table 6. Description of Phenotypes Collected during the Study.** Descriptions for the collected phenotypes and references to literature that provide extensive definition of collection methods. Analyzed phenotypes were collected during months 13-31 of experimental phase.

| **Category** | **Phenotype** | **Brief description** |
| --- | --- | --- |
| **Blood Pressure (Month 26)** | Systolic BP  Diastolic BP  Mean BP  Pulse Wave Velocity | Systolic blood pressure by tail high definition oscillometry (Shively et al. 2020 doi:10.1016/j.ynstr.2020.100254)  Diastolic blood pressure by tail high definition oscillometry (Shively et al. 2020 doi:10.1016/j.ynstr.2020.100254)  Mean arterial blood pressure by tail high definition oscillometry (Shively et al. 2020 doi:10.1016/j.ynstr.2020.100254)  Pulse wave velocity by tail high definition oscillometry (Shively et al. 2020 doi:10.1016/j.ynstr.2020.100254) |
| **Stress** | Acute Stress Cortisol  ACTH Challenge Cortisol | Month 29 cortisol area under  the curve in response to the stress from brief social separation (Shively et al. 2020 doi:10.1016/j.ynstr.2020.100254)  Month 30 cortisol area under the curve in response to adrenocorticotropin (ACTH) challenge (Shively et al. 2020 doi:10.1016/j.ynstr.2020.100254) |
| **Heart Rate Variability (HRV) (Month 30)** | Day %VLF HR Oscillations  Day %LF HR Oscillations  Day %HF HR Oscillations  Day LF/HF HR Oscillations  Day SDNN  Day RMSSD  Day Mean HR  Night %VLF HR Oscillations  Night %LF HR Oscillations  Night %HF Oscillations  Night LF/HFHR Oscillations  Night SDNN  Night RMSSD | Percentage of very low frequency (VLF) oscillations during the day at 1600-1800 hours (Shively et al. 2020 doi:10.1016/j.ynstr.2020.100254)  Percentage of low frequency (LF) oscillations during the day at 1600-1800 hours (Shively et al. 2020 doi:10.1016/j.ynstr.2020.100254)  Percentage of high frequency (HF) oscillations during the day at 1600-1800 hours (Shively et al. 2020 doi:10.1016/j.ynstr.2020.100254)  Ratio of LF/HF oscillations during the day 1600-1800 hours (Shively et al. 2020 doi:10.1016/j.ynstr.2020.100254)  Mean standard deviation of normal R-to-R intervals (SDNN) during the day at 1600- 1800 hours (Shively et al. 2020 doi:10.1016/j.ynstr.2020.100254)  Average root mean square of successive differences between R-R intervals (RMSSD) during the day at 1600-1800  Hours (Shively et al. 2020 doi:10.1016/j.ynstr.2020.100254)  Mean heart rate during the day at 1600-1800 hours (Shively et al. 2020 doi:10.1016/j.ynstr.2020.100254)  Percentage of VLF oscillations during the night at 0100-0300 hours (Shively et al. 2020 doi:10.1016/j.ynstr.2020.100254)  Percentage of LF oscillations during the night at 0100-0300 hours (Shively et al. 2020 doi:10.1016/j.ynstr.2020.100254)  Percentage of high  frequency (HF) oscillations  during the night at 0100-0300 hours (Shively et al. 2020 doi:10.1016/j.ynstr.2020.100254)  Ratio of LF/HF oscillations during the night at 0100-0300 hours (Shively et al. 2020 doi:10.1016/j.ynstr.2020.100254)  Mean standard deviation of normal R-to-R intervals (SDNN) during the night at 0100- 0300 hours (Shively et al. 2020 doi:10.1016/j.ynstr.2020.100254)  Average root mean square of successive differences between R-R intervals (RMSSD) during the night at 0100- 0300 hours (Shively et al. 2020 doi:10.1016/j.ynstr.2020.100254) |
| **Morphometrics** | BMI  % Whole Body Fat  Liver Attenuation | Body mass index  (Body weight/(body length [meters])^2^), measured at month 31 (Shively et al. 2019 doi:10.1002/oby.22436)  Per cent whole body fat measured by CT scan at month 27 (Shively et al. 2019 doi:10.1002/oby.22436)  Mean Liver attenuation measured by CT scan at month 27 (Shively et al. 2019 doi:10.1002/oby.22436) |
| **Insulin (Month 26)** | IV GTT Insulin AUC | Insulin areas under the curve between minutes 10-40 post IV glucose in an intravenous glucose tolerance test (Shively et al. 2019 doi:10.1002/oby.22436) |
| **Coronary artery atherosclerosis (Necropsy)** | LAD Atherosclerosis  Coronary Artery Atherosclerosis | Left anterior descending coronary artery atherosclerosis extent (Shively et al. 2015 doi: 10.1097/psy.0000000000000163)  Mean coronary artery  atherosclerosis extent (Shively et al. 2015 doi: 10.1097/psy.0000000000000163) |
| **Ovarian Function (Month 16-27)** | Menstrual Cycle Length  Mean Peak Progesterone | Average length of cycles  during experimental phase, measured in days (Frye et al. 2023 doi: 10.1016/j.psyneuen.2023.106107)  Mean Peak progesterone per  cycle during experimental  phase (Frye et al. 2023 doi: 10.1016/j.psyneuen.2023.106107) |

**Supplementary Table 7.** Spearman's correlations between top DEGs in the iliac arteries and physical, behavioral, and physiological phenotypes collected during the experimental phase adjusted for diet. (Bold font denotes p values < 0.05.)

**Supplementary Table 8.** Spearman's correlations between top DEGs in the carotid arteries and physical, behavioral, and physiological phenotypes collected during the experimental phase adjusted for relative rank. (Bold font denotes p values < 0.05.)
